# Assessing the impact of hatching system and body weight on the growth performance, caecal short-chain fatty acids, and microbiota composition and functionality in broilers

**DOI:** 10.1186/s42523-024-00331-6

**Published:** 2024-07-24

**Authors:** Muhammad Zeeshan Akram, Ester Arévalo Sureda, Luke Comer, Matthias Corion, Nadia Everaert

**Affiliations:** 1 Nutrition and Animal-Microbiota Ecosystems Laboratory, Department of Biosystems, KU Leuven, Heverlee, 3000 Belgium; 2 Precision Livestock and Nutrition Laboratory, Gembloux Agro-Bio Tech, TERRA Teaching and Research Centre, University of Liège, Gembloux, B-5030 Belgium

**Keywords:** Poultry, Growth performance, Flock uniformity, Gut health, Microbiome

## Abstract

**Background:**

Variations in body weight (BW) remain a significant challenge within broiler flocks, despite uniform management practices. Chicken growth traits are influenced by gut microbiota, which are in turn shaped by early-life events like different hatching environments and timing of first feeding. Chicks hatched in hatcheries (HH) experience prolonged feed deprivation, which could adversely impact early microbiota colonization. Conversely, hatching on-farm (HOF) allows early feeding, potentially fostering a more favorable gut environment for beneficial microbial establishment. This study investigates whether BW differences among broilers are linked to the disparities in gut microbiota characteristics and whether hatching systems (HS) impact the initial microbial colonization of broilers differing in BW, which in turn affects their growth patterns. Male Ross-308 chicks, either hatched in a hatchery or on-farm, were categorized into low (LBW) and high (HBW) BW groups on day 7, making a two-factorial design (HS × BW). Production parameters were recorded periodically. On days 7, 14, and 38, cecal volatile fatty acid (VFA) and microbiota composition and function (using 16 S rRNA gene sequencing and PICRUSt2) were examined.

**Results:**

HOF chicks had higher day 1 BW, but HH chicks caught up within first week, with no further HS-related performance differences. The HBW chicks remained heavier attributed to higher feed intake rather than improved feed efficiency. HBW group had higher acetate concentration on day 14, while LBW group exhibited higher isocaproate on day 7 and isobutyrate on days 14 and 38. Microbiota analyses revealed diversity and composition were primarily influenced by BW than by HS, with HS having minimal impact on BW-related microbiota. The HBW group on various growth stages was enriched in VFA-producing bacteria like unclassified *Lachnospiraceae*,* Alistipes* and *Faecalibacterium*, while the LBW group had higher abundances of *Lactobacillus*, *Akkermansia* and *Escherichia-Shigella*. HBW microbiota presented higher predicted functional potential compared to the LBW group, with early colonizers exhibiting greater metabolic activity than late colonizers.

**Conclusions:**

Despite differences in hatching conditions, the effects of HS on broiler performance were transient, and barely impacting BW-related microbiota. BW variations among broilers are likely linked to differences in feed intake, VFA profiles, and distinct microbiota compositions and functions.

**Supplementary Information:**

The online version contains supplementary material available at 10.1186/s42523-024-00331-6.

## Background

Intensive genetic selection and optimized management practices in modern broiler production have led to remarkable improvements in growth rate and feed efficiency [1]. Despite all these efforts, achieving consistent body weight (BW) uniformity within a broiler flock at market age remains a persistent challenge [2]. Variation in final BW, as indicated by the coefficient of variation (CV), is a critical concern in the broiler industry due to its association with reduced feed efficiency, increased mortality, poor growth rates, and higher rates of market rejection [3–5]. Despite persistent research efforts, the underlying mechanisms that contribute to weight heterogeneity within a broiler flock are not yet conclusively understood.

The gut microbiota has emerged as a significant factor influencing the physiological characteristics and performance of chickens [6]. The resident gut microbiota possess the capacity to extract energy from otherwise indigestible feed components via fermentation, producing high-energy by-products such as short-chain fatty acids (SCFAs) [7]. These microbial-derived metabolites can modulate various host physiological functions, including metabolism, immunity, and intestinal barrier integrity [8].

The composition and functional capabilities of the intestinal microbiota have been extensively investigated for their potential links to broiler growth performance, however, the results have been varied and contradictory. Han et al. [9] reported a negative correlation between microbial diversity in the caecum and BW, while Abdel-Kafy et al. [10] found no differences in microbial diversity between chickens varying in growth rate. Certain bacterial genera considered beneficial, such as *Bacteroides* and *Lactobacillus*, have been associated with high weight gain and improved growth [11], but *Lactobacillus* has also been negatively correlated with BW in both the ileum and caecum [12]. Additionally, the Proteobacteria genus *Escherichia–Shigella* has been negatively correlated, while the Firmicutes genus *Clostridium coccoides* has been positively correlated with weight gain [13]. These discrepancies may be attributed to differences in chicken genotypes, sex, geographical regions, rearing conditions, sampling time points, and intestinal sites analyzed.

A few studies have comprehensively examined distinct gut microbial signatures and functional profiles in broilers exhibiting extreme differences in BW. A recent investigation by Lundberg et al. [14] identified taxa such as *Lachnospiraceae*, *Faecalibacterium*, and *Butyricicoccus* to be enriched in high body weight (HBW) broilers, while *Akkermansia* and Escherichia-Shigella were more abundant in low body weight (LBW) counterparts on day 37. Furthermore, Lee et al. [15] found that higher abundances of *Shuttleworthia* and *Faecalibacterium* in HBW male chickens on day 35 post-hatch. However, the majority of studies have focused on a single time point, typically near or at market age, limiting the understanding of dynamic gut microbial changes during early life.

First gut microbiota colonization has been reported to influence microbiome succession and host growth in later stages [16]. While the influence of early life experiences on broiler development has been acknowledged, limited research has explored the specific effects of hatching conditions on broiler microbiota and subsequent growth patterns. Traditionally, broiler chicks hatch in artificial incubators under a relatively sterile environment (egg and incubator sterilization) without maternal-offspring interaction [13]. Additionally, hatchery-hatched (HH) chicks face delayed access to feed and water due to long hatching windows and hatchery logistic procedures [17]. This implies the lack of proper early exposure to microorganisms particularly to those with beneficial effects, increasing the likelihood of exposure to environmental pathogens [18].

Alternatively, hatching on-farm (HOF) involves the transportation of embryonated eggs to the broiler house on day 18 of incubation, allowing immediate access to feed and water for chicks at hatching [17]. This approach has the potential to foster a more favorable environment for early gut development and beneficial microbiota colonization. Since chickens on farms encounter a wide variety of microorganisms present in litter, feed, water, and air, thus the conditions during the hatching process can play a crucial role in shaping the initial colonization of the gut microbiota. For example, it was highlighted that chicks originating from hatcheries often exhibit delayed and highly variable development of their gut microbiota [19]. This variability is anticipated to be reduced in chicks with early access to feed, as demonstrated by the observed similarities between the microbiota of their diet and that of their intestines [20].

To delve deeper into the aforementioned aspects, we designed a study to investigate the intricate relationships between BW and caecal microbiota dynamics and the impact of different hatching systems (HS) on microbial signatures in birds with different BWs. The aim in this study was to explore whether broilers with varying BWs have differences in performance indices, caecal volatile fatty acids (VFAs), microbiota community structures, and predicted functionality on days 7, 14, and 38 under shared management conditions. Thereby, we extend beyond existing studies that majorly focus on single time points, particularly at slaughter age. Consequently, we characterized crucial changes in intestinal microbiota also during early life stages that influence the succession of gut microbiota and subsequent growth trajectories. We also investigated how HS may differentially impact initial microbiota colonization in broilers with different weights and shape their post-hatch microbiota development and growth patterns.

## Results

### Growth performance

HS significantly influenced BW at placement (*P* < 0.05), with HOF chicks exhibiting higher BW on day 1 (45.1 ± 3.14 g, *n* = 454) compared to HH chicks (42.2 ± 2.92 g, *n* = 454). This difference in BW between HS disappeared by day 7, and the chicks hatched in either system no longer differed in any performance indices thereafter (Table 1, *P* > 0.05). The chicks from both HS were categorized into LBW and HBW groups on day 7, revealing a significant difference in BW (*P* < 0.05). From day 7 onwards, there was no point at which chicks in the LBW group were able to catch up and they consistently demonstrated lower BW (*P* < 0.05) on days 14, 28, and 38 compared to chicks in the HBW group. Similarly, the average daily gain (ADG) was lower (*P* < 0.001) in chicks of the LBW group than in those in the HBW group except during 29–38 days. Lower initial BW was accompanied by a lower feed intake, and chicks in the LBW group demonstrated lower average daily feed intake (ADFI) (*P* < 0.05) than chicks in the HBW group during 7–14 days, 15–28 days, 29–38 days, and 7–38 days, respectively. The feed conversion ratio (FCR) was lower (*P* = 0.021) in the LBW group than in the HBW group during the overall period (7–38 days). The coefficient of variation (CV) for BW [CV(%) = flock heterogeneity] was lower in the HBW group on days 14, 28, and 38 than in the LBW group (*P* < 0.05). There was no interaction (*P* > 0.05) between HS and BW for any growth performance measurements. Finally, no differences in mortality (*P* > 0.05) were observed between LBW and HBW birds of either HS over the 38-day period (data not shown).

**Table 1:** Growth performance of low (LBW) and high (HBW) body weight broilers hatched in the hatchery (HH) or on-farm (HOF)

^1^Items	^2^Groups (*n* = 7 pen/group)					^3^*P*-values		
	HH-LBW	HH-HBW	HOF-LBW	HOF-HBW	SD	HS	BW	HS × BW
BW, g								
Day 7	166^b^	206^a^	159^b^	211^a^	23.7	0.698	**< 0.001**	0.268
Day 14	451^b^	572^a^	446^b^	563^a^	62.9	0.364	**< 0.001**	0.724
Day 28	1716^b^	2025^a^	1701^b^	2012^a^	160.6	0.876	**< 0.001**	0.426
Day 38	2962^b^	3259^a^	2946^b^	3248^a^	177.1	0.623	**< 0.001**	0.770
ADG, g BW/day								
7–14 days	41.1^b^	52.3^a^	40.7^b^	51.5^a^	5.82	0.362	**< 0.001**	0.808
15–28 days	90.2^b^	103.3^a^	89.7^b^	102.9^a^	7.65	0.746	**< 0.001**	0.822
29–38 days	125.3	124.2	124.6	123.8	9.40	0.881	0.796	0.974
7–38 days	94.3^b^	103.7^a^	93.6^b^	103.4^a^	5.81	0.701	**< 0.001**	0.891
ADFI, g feed/day								
7–14 days	47.0^b^	57.5^a^	46.5^b^	58.9^a^	6.67	0.740	**< 0.001**	0.499
15–28 days	117.2^b^	137.0^a^	117.1^b^	136.2^a^	11.21	0.827	**< 0.001**	0.903
29–38 days	184.7^b^	198.9^a^	189.9^b^	200.2^a^	13.03	0.470	**0.012**	0.682
7–38 days	123.1^b^	139.0^a^	124.7^b^	139.4^a^	9.31	0.630	**< 0.001**	0.779
FCR, g feed/g BW								
7–14 days	1.16	1.16	1.19	1.20	0.198	0.336	0.858	0.933
15–28 days	1.34	1.36	1.36	1.39	0.206	0.479	0.464	0.958
29–38 days	1.53	1.61	1.54	1.66	0.261	0.732	0.056	0.522
7–38 days	1.40^b^	1.45^a^	1.42^b^	1.45^a^	0.143	0.621	**0.021**	0.366
CV in BW (%)								
Day 7	4.9	4.8	5.0	4.9	0.28	0.679	0.102	0.956
Day 14	6.9^b^	5.2^a^	7.4^b^	5.7^a^	1.72	0.321	**0.007**	0.892
Day 28	8.7^b^	6.8^a^	9.2^b^	7.1^a^	1.37	0.868	**≤ 0.001**	0.168
Day 38	12.9^b^	7.9^a^	13.8^b^	8.2^a^	3.08	0.472	**≤ 0.001**	0.700

^1^BW: body weight; ADG: average daily gain; ADFI: average daily feed intake; FCR: feed conversion ratio; CV: coefficient of variation (inversely related to flock uniformity)

^2^HH-LBW: hatchery-hatched low BW group (*n* = 7 pens), HH-HBW: hatchery-hatched high BW group (*n* = 7 pens), HOF-LBW: hatched on-farm low BW group (*n* = 7 pens), HOF-HBW: hatched on-farm high BW group (*n* = 7 pens)

^3^HS: main effect of hatching system; BW: main effect of BW; HS × BW: interaction between HS and BW. Except for BW data, the pen was considered as an experiment unit

Data are presented as mean and pooled standard deviation (SD)

Values in a row with different superscript letters (^a, b^) indicate significant difference at *P* < 0.05 (Two-way ANOVA with post hoc Tukey HSD)

### Caecal microbiota

A total of 3,763,252 reads obtained from 120 samples were used in the microbiota analysis, resulting in an average of 31,360 reads per sample with a SD of 11,410 reads (Range: minimum = 11,711 and maximum = 60,170). To ensure uniformity in the α-diversity analysis, the sample with the minimum number of reads (11,711) was established as the cut-off threshold for rarefying all samples.

#### Α-diversity

**Fig. 1:**
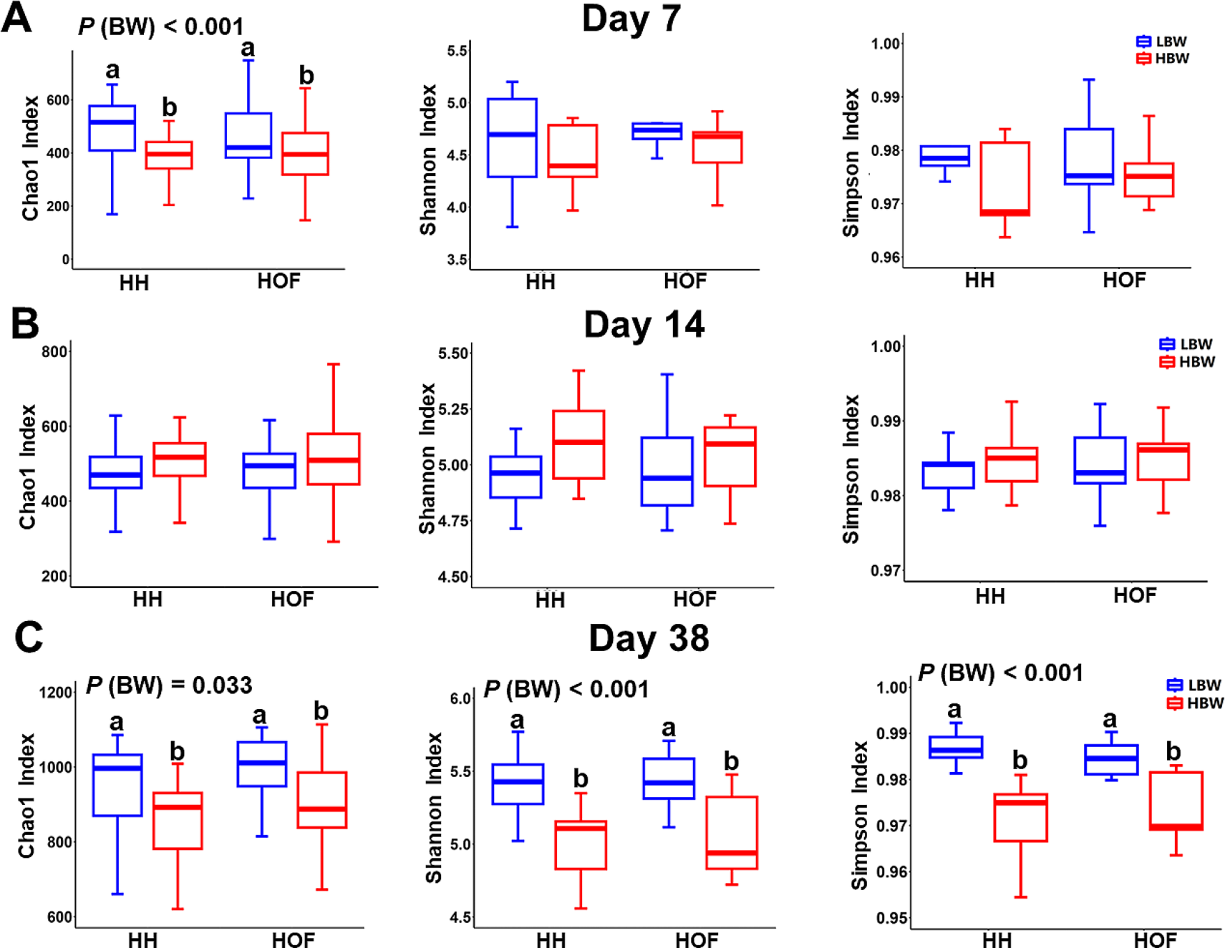
α-diversity measures (Chao1, Shannon and Simpson index) of the caecal microbiota of low (LBW) and high (HBW) body weight chickens hatched in the hatchery (HH) and on-farm (HOF) systems on day 7 (**A**), day 14 (**B**), and day 38 (**C**). Individually sampled chickens were considered as experimental unit (*n* = 10 per group). α-diversity measures were evaluated by two-way ANOVA and significant differences were only found for BW, indicated with different letters with *P* (BW) < 0.05

α-diversity metrics were not affected by HS at any time point (Fig. 1, *P* > 0.05). However, BW significantly influenced α-diversity, with higher Chao1 index values on day 7 (*P* < 0.001) and day 38 (*P* = 0.033) and increased Shannon and Simpson index values on day 38 (*P* < 0.001) in chicks of the LBW group than those in the HBW group. No interaction between HS and BW for α-diversity was deemed significant at any time point (*P* > 0.05).

#### β-diversity

**Fig. 2:**
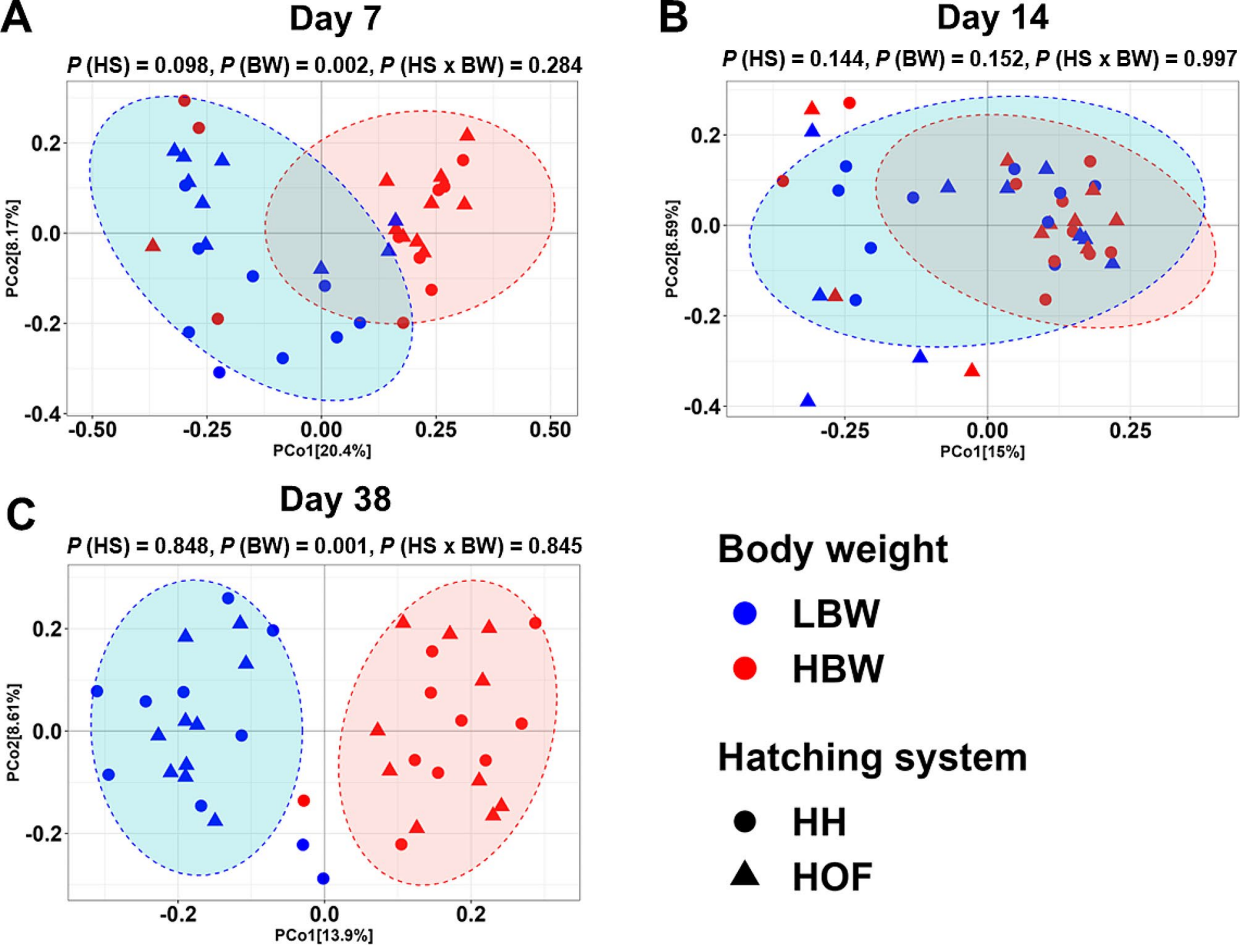
Principal coordinate analysis (PCoA) for log-transformed Bray–Curtis dissimilarity matrices of caecal microbiota of low (LBW) and high (HBW) body weight (BW) chickens hatched in the hatchery (HH) or on-farm (HOF) on day 7 (**A**), day 14 (**B**), and day 38 (**C**). The color of the dots represents BW groups and their shapes represent the hatching system (HS). Individually sampled chickens were considered as experimental unit (*n* = 10 per group). Multivariate effects of HS and BW on β-diversity were evaluated by non-parametric permutational multivariate analysis of variance (PERMANOVA) and significant differences were only found for BW. The *P*-values for HS, BW, and their interaction are indicated with different letters with *P* (BW), *P* (HS), and *P* (HS × BW), respectively

β-diversity analysis using Bray-Curtis distances did not show any differences for the HS at any time point (Fig. 2). However, β-diversity was significantly different between LBW and HBW groups on days 7 and 38, with two distinct clusters based on the BW groups were observed (*P* = 0.002 and R^2^ = 0.042 for day 7, and *P =* 0.001 and R^2^ = 0.027 for day 38, Fig. 2A and C). The interaction between HS and BW for β-diversity was found to be non-significant throughout the study (*P* > 0.05).

### Core microbiota composition

**Fig. 3:**
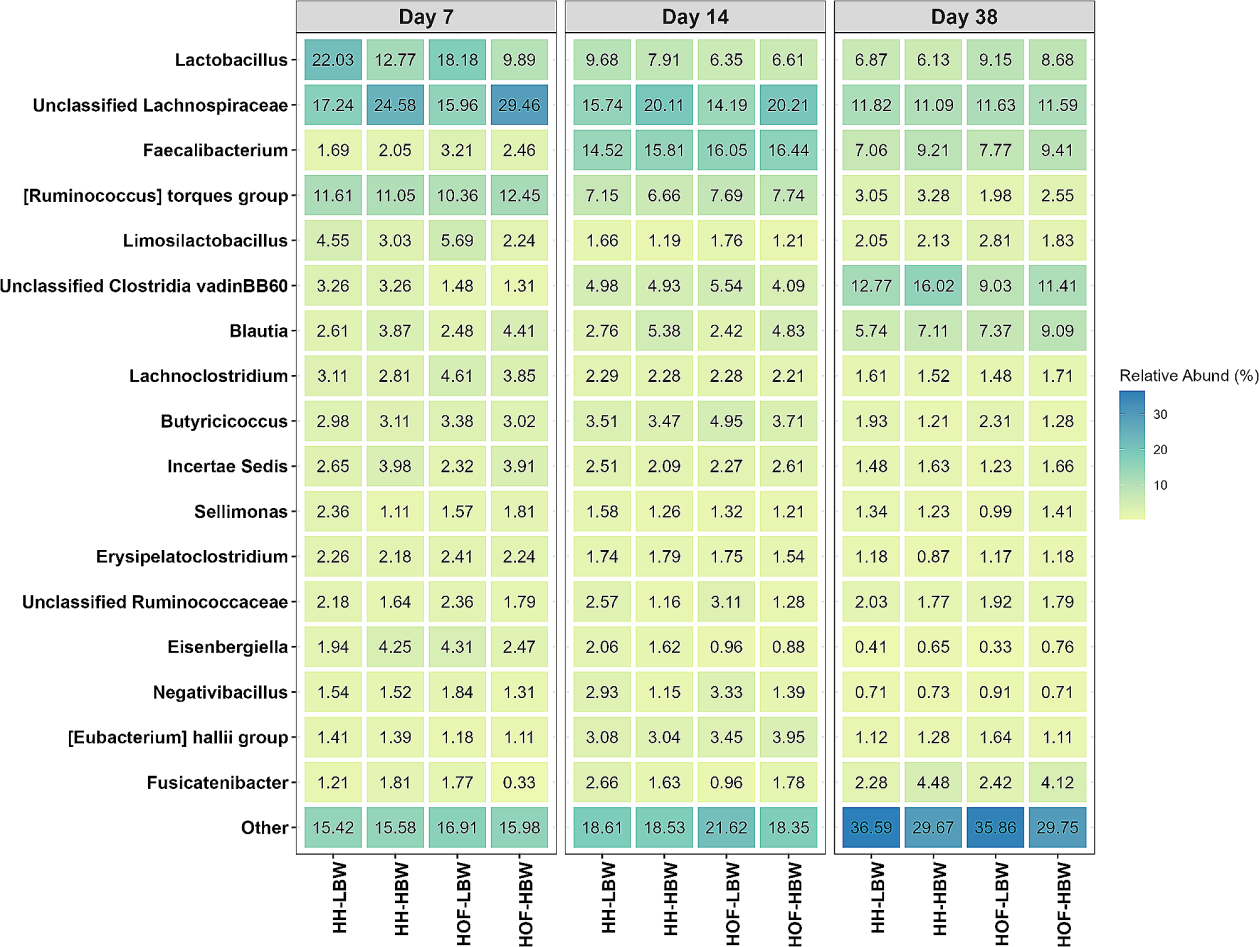
Relative abundance of caecal bacterial genera in low (LBW) and high (HBW) body weight chickens hatched in the hatchery (HH) or on-farm (HOF) on days 7, 14, and 38. Values indicate the mean relative abundance (%) of the top 17 genera (Y-axis). Individually sampled chickens were considered as experimental unit (*n* = 10 per group)

Compositional analysis consistently identified Firmicutes as the predominant phylum in chickens from both HS throughout the study (Additional file 1, Table S1). This phylum represented ∼ 99% of the total relative abundance on day 7, ∼ 97% on day 14, and ∼ 93% on day 38. Following Firmicutes, Bacteroidota, and Proteobacteria emerged as the next dominant phyla across all three time points, with Cyanobacteria joining in notable relative abundance by day 38. At the genus level, HH and HOF chickens exhibited a distinctive dominance of unclassified *Lachnospiraceae*, and *Lactobacillus* on day 7 (16–30% and 10–22% respectively), followed by the *[Ruminococcus] torques* group (10–13%) and *Lachnoclostridium* (3–5%, Fig. 3**)**. By day 14, the dominant genera included unclassified *Lachnospiraceae* (14–20%) and *Faecalibacterium* (15–17%), along with *Lactobacillus* and the *[Ruminococcus] torques* group at 6–10% and 7–8%, respectively. By day 38, the most predominant genera were the unclassified *Clostridia vadinBB60* group (9–16%) and unclassified *Lachnospiraceae* (11–12%), followed by *Faecalibacterium* (7–9%), *Lactobacillus* (6–9%), and *Blautia* (6–9%).

### Differential abundance of bacteria

**Fig. 4:**
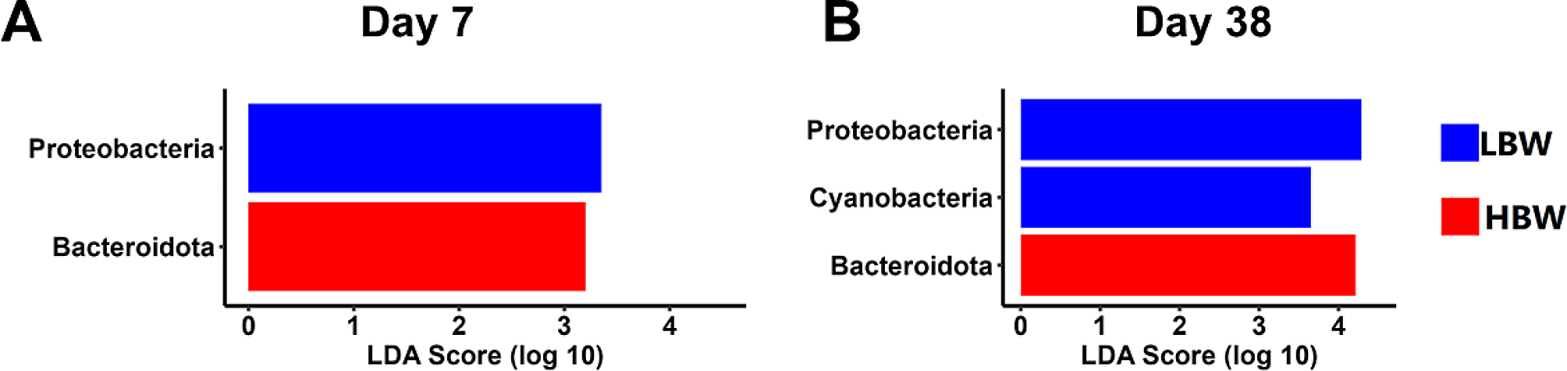
Differential abundance of phyla (FDR < 0.05 and |LDA| > 2 ) in samples from low (LBW) and high (HBW) body weight chickens on day 7 (**A**), and day 38 (**B**). No differences were observed on day 14

Linear discriminant analysis (LDA) effect size (LEfSe) analysis was used to determine the differential phylum abundance between groups using a false discover rate (FDR) cut-off value of 0.05 with a minimum LDA score of 2. At the phylum level, no differences were observed for HS (HH vs. HOF) or the HS × BW interaction. BW-dependent differences were observed at the phylum level on days 7 and 38 (Fig. 4A and B). Bacteroidota phylum was differentially enriched in HBW chickens on days 7 and 38, while Proteobacteria was more abundant in LBW chickens on days 7 and 38 along with Cyanobacteria on day 38 (FDR < 0.05). No differential abundance was found at the phylum level on day 14.

**Fig. 5:**
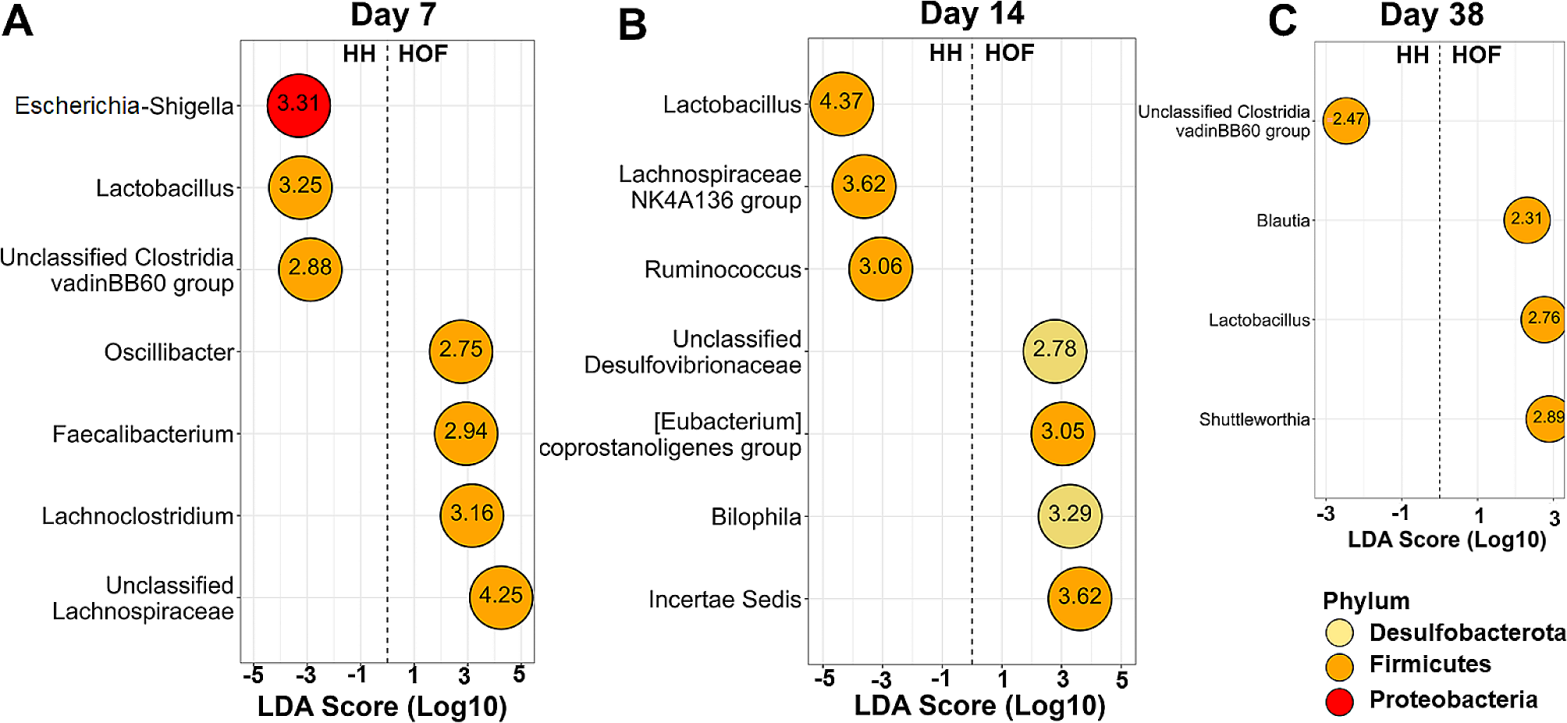
LEfSe results of differentially abundant genera for chicks hatched in the hatchery (HH) vs. on-farm (HOF) on day 7 (**A**), day 14 (**B**), and day 38 (**C**). Only genera with an FDR ≤ 0.05 and an absolute value of LDA > 2 are presented. The left-hand side of each plot indicates bacterial genera enriched in HH chickens, and on the right-hand side, bacterial genera enriched in HOF chickens

The differential abundance of bacterial genera between HH and HOF chickens was determined on days 7, 14, and 38 using LEfSe analysis using an FDR cut-off value of 0.05 with a minimum LDA score of 2. (Fig. 5). On day 7, HH chicks exhibited enriched *Escherichia-Shigella*, *Lactobacillus*, and unclassified *Clostridia vadinBB60 group* (FDR < 0.05), while HOF chicks showed greater relative abundance of unclassified *Lachnospiraceae*, *Lachnoclostridium*, *Faecalibacterium*, and *Oscillibacter* (Fig. 5A). By day 14, HH chicks were enriched in *Lactobacillus*, *Lachnospiraceae NK4A136 group*, and *Ruminococcus*, while HOF chicks had a higher abundance of *Incertae Sedis*, *Bilophila*, and unclassified *Desulfovibrionaceae* (Fig. 5B). By day 38, microbiota differences between HS substantially reduced, with only four genera showing differential abundance. The HH chicks had an higher abundance of unclassified *Clostridia vadinBB60 group*, while HOF chicks had a higher abundance of *Shuttleworthia*, *Lactobacillus*, and *Blautia* (Fig. 5C).

**Fig. 6:**
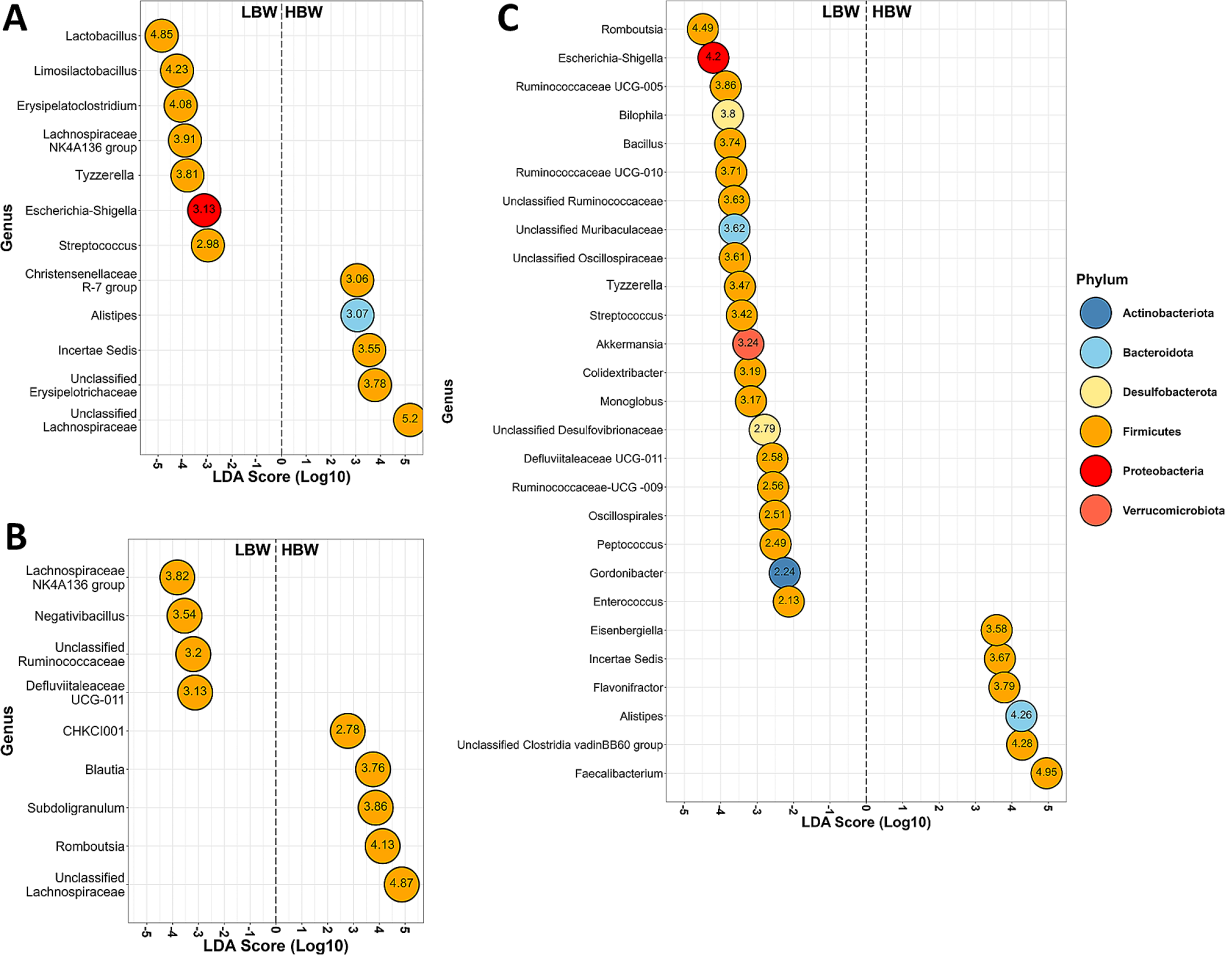
LEfSe results of the differential abundance of genera in the caecal microbiota of chickens with low (LBW) and high (HBW) body weight on day 7 (**A**), day 14 (**B**), and day 38 (**C**). Only genera with an FDR ≤ 0.05 and with an absolute value of LDA > 2 are presented. The lefthand side of plots indicates bacterial genera enriched in LBW chickens, and on the right hand side, bacterial genera enriched in HBW chickens

Differential abundance of bacterial genera between BW groups was also determined on days 7, 14, and 38 using LEfSe analysis (Fig. 6). On day 7, the LBW group showed enrichment in seven genera, including *Escherichia-Shigella*, *Streptococcus*, *Limosilactobacillus* and *Lactobacillus*, while the HBW group had higher abundance in five genera, including unclassified *Lachnospiraceae*,* Christensenellaceae R-7 group*, and *Alistipes* (Fig. 6A). By day 14, LBW group were significantly enriched with four genera, including *Lachnospiraceae NK4A136* group, unclassified *Ruminococcaceae* and *Negativibacillus*, while the HBW group exhibited increased abundance in five genera, such as unclassified *Lachnospiraceae*, *Subdoligranulum*, *Romboutsia*, and *Blautia* (Fig. 6B). The differences in microbiota composition between BW groups increased over time, with the LBW group on day 38 showing differential enrichment of 21 genera, including *Escherichia-Shigella*, *Enterococcus*, *Bilophila*, *Streptococcus*, and *Akkermansia*, compared to the HBW group, which exhibited increased abundances of six genera, including *Faecalibacterium*, unclassified *Clostridia vadinBB60 group*, and *Alistipes* (Fig. 6C). A few interactions between HS and BW were observed for microbiota differential abundance analysis (Additional file 1, Fig. S1). Specifically, *Lactobacillus* was enriched in HH-LBW chicks on day 7, while unclassified *Lachnospiraceae* was enriched in HOF-HBW chicks (Fig. S1 A). Two genera were differentially abundant on day 38, with HOF-LBW birds having a higher abundance of unclassified *Desulfovibrioceace*, while HH-HBW chickens had an overabundance of unclassified *Clostridia vadinBB60 group* (Fig. S1 B). No significant differences for HS × BW interaction were observed on day 14.

### Concentration of volatile fatty acids in caecum

HS did not significantly influence VFAs, but BW and the interaction between HS and BW affected their concentrations on various days (Table 2). On day 7, Isocapraote production was higher in the LBW group. The HBW group demonstrated higher acetate and total SCFA concentrations on day 14, while the LBW exhibited higher levels of isobutyrate on days 14 and 38, as well as increased isovalerate and total branched-chain fatty acids (BCFAs) on day 14. Furthermore, propionate and total BCFA concentrations on day 38 tended to be higher in the LBW group. An interaction between HS and BW was observed on day 7, with the HH-LBW group exhibiting higher valerate concentrations. A tendency towards an interaction between HS and BW was noted, with acetate and total SCFA levels tending to be higher in the HOF-HBW group on day 7, and valerate levels tending to be higher on day 14.

**Table 2:** Caecal volatile fatty acid (VFA) concentrations (mM/g wet digesta) of low (LBW) and high (HBW) body weight broiler chickens hatched in the hatchery (HH) or on-farm (HOF)

^1^Items	^2^Groups (*n* = 10/group)					^3^*P*-values		
	HH-LBW	HH-HBW	HOF-LBW	HOF-HBW	SD	HS	BW	HS × BW
Day 7								
Acetate	200.5	155.8	182.9	249.6	73.61	0.212	0.713	0.074
Propionate	6.5	4.8	5.1	6.0	3.61	0.948	0.788	0.429
Butyrate	25.9	21.1	24.5	28.9	8.31	0.393	0.950	0.208
Valerate	1.39^a^	0.74^bc^	0.56^c^	0.94^b^	0.596	0.212	0.566	**0.047**
Caproate	0.04	0.02	0.01	0.03	0.022	0.134	0.540	0.109
Total SCFAs	234.6	182.6	213.2	285.7	79.02	0.210	0.750	0.068
Isobutyrate	0.96	0.77	0.87	0.98	0.358	0.349	0.806	0.734
Isovalerate	0.57	0.35	0.53	0.61	0.214	0.215	0.411	0.107
Isocaproate	0.26^a^	0.19^b^	0.22^a^	0.21^b^	0.041	0.332	**0.019**	0.095
Total BCFAs	1.79	1.31	1.62	1.79	0.552	0.528	0.533	0.185
Day 14								
Acetate	185.2^b^	217.3^a^	177.5^b^	207.8^a^	45.31	0.545	**0.032**	0.955
Propionate	10.9	11.4	10.8	13.4	5.79	0.648	0.423	0.571
Butyrate	40.3	40.6	37.6	39.8	14.78	0.551	0.641	0.683
Valerate	2.21	2.14	1.76	2.30	0.641	0.432	0.123	0.077
Caproate	0.07	0.06	0.06	0.07	0.031	0.728	0.851	0.356
Total SCFAs	238.8^b^	277.4^a^	225.4^b^	263.4^a^	53.79	0.524	**0.041**	0.875
Isobutyrate	1.42^a^	0.97^b^	1.16^a^	0.87^b^	0.520	0.247	**0.022**	0.588
Isovalerate	1.18^a^	0.80^b^	1.09^a^	0.91^b^	0.440	0.932	**0.049**	0.489
Isocaproate	0.18	0.15	0.17	018	0.093	0.688	0.697	0.603
Total BCFAs	1.25^a^	0.87^b^	1.09^a^	0.88^b^	0.427	0.586	**0.028**	0.501
Day 38								
Acetate	212.2	227.1	209.1	218.3	88.56	0.840	0.661	0.922
Propionate	14.1	12.4	14.8	11.5	4.86	0.860	0.079	0.860
Butyrate	41.8	43.5	42.8	42.9	15.7	0.963	0.850	0.878
Valerate	1.91	2.05	2.17	1.91	0.586	0.741	0.743	0.302
Caproate	0.07	0.06	0.08	0.06	0.042	0.634	0.242	0.725
Total SCFAs	270.5	285.2	269.1	274.8	102.0	0.862	0.762	0.895
Isobutyrate	1.63^a^	1.41^b^	1.67^a^	1.34^b^	0.438	0.927	**0.048**	0.710
Isovalerate	1.46	1.42	1.43	1.14	0.435	0.237	0.236	0.392
Isocaproate	0.23	0.25	0.26	0.24	0.029	0.335	0.832	0.167
Total BCFAs	3.33	3.07	3.35	2.72	0.810	0.511	0.086	0.475

^1^SCFAs: short-chain fatty acids (Acetate, propionate, butyrate, valerate, and caproate); BCFAs: branched-chain fatty acids (Isobutyrate, Isovalerate, and Isocaproate)

^2^HH-LBW: hatchery-hatched low BW group (*n* = 10), HH-HBW: hatchery-hatched high BW group (*n* = 10), HOF-LBW: hatched on-farm low BW group (*n* = 10), HOF-HBW: hatched on-farm high BW group (*n* = 10). Individually sampled chickens were considered as experimental unit

^3^HS: main effect of hatching system; BW: main effect of body weight; HS × BW: interaction between HS and BW.

Data are presented as mean and pooled standard deviation (SD).

Values in a row with different superscript letters (^a, b^) indicate significant difference at *P* < 0.05 (Two-way ANOVA with post hoc Tukey HSD)

**Fig. 7:**
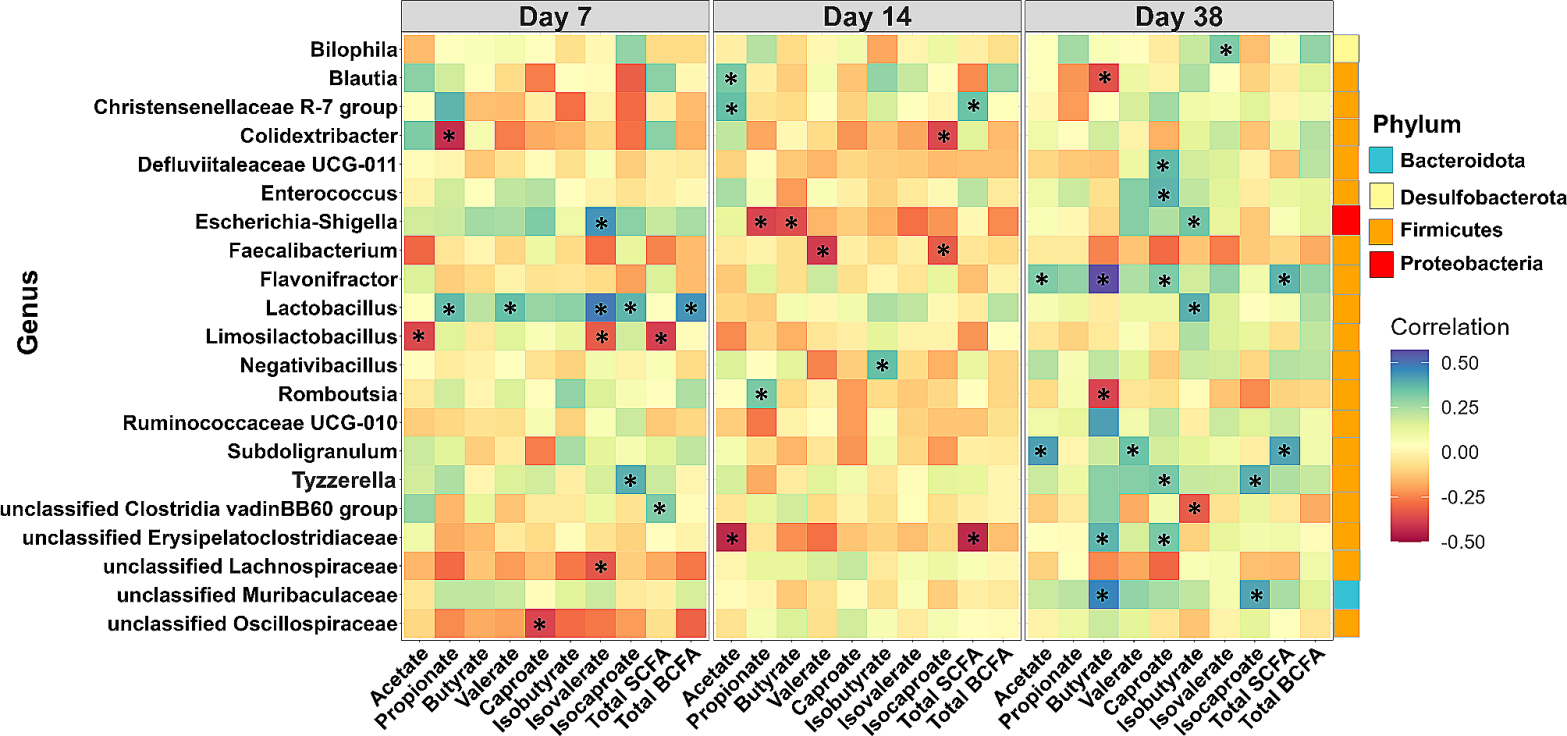
Heatmap of Spearman correlation on days 7, 14 and 38 between caecal VFA concentrations and relative abundance of bacterial genera that were differentially enriched among BW groups based on the LEfSe analysis. Bacterial genera are color-labeled with their corresponding phyla (right side of the figure). Correlations with an FDR < 0.05 and |R| > 0.33 were considered significant and were indicated with an asterisk

Figure 7 shows Spearman correlations between VFA concentrations and differentially enriched bacterial genera across 3 time points, with the highest number of significant (positive) correlations found on day 38. Correlations with an FDR < 0.05 and |R| > 0.33 were considered significant and were indicated with an asterisk. *Blautia* correlated positively with acetate concentration on day 14 but negatively correlated with butyrate on day 38. *Lactobacillus* was positively correlated with propionate, valerate, isovalerate, isocaproate and total BCFAs on day 7, and isobutyrate on day 38. *Limosilactobacillus* showed a negative correlation with acetate, isovalerate, and total SCFAs on day 7. *Negativibacillus* positively correlated with isobutyrate on day 14. *Romboutsia* showed a positive correlation with propionate on day 14, and a negative correlation with butyrate on day 38. *Christensenellaceae R-7* group positively correlated with propionate and total SCFAs on day 14 *Escherichia-Shigella* correlated positively with isovalerate on day 7, negatively with propionate and butyrate on day 14, and positively with isobutyrate on day 38. *Flavonifractor* positively correlated with acetate, butyrate, caproate, and total SCFA production on day 38.

### Microbiota functional profiling

**Fig. 8:**
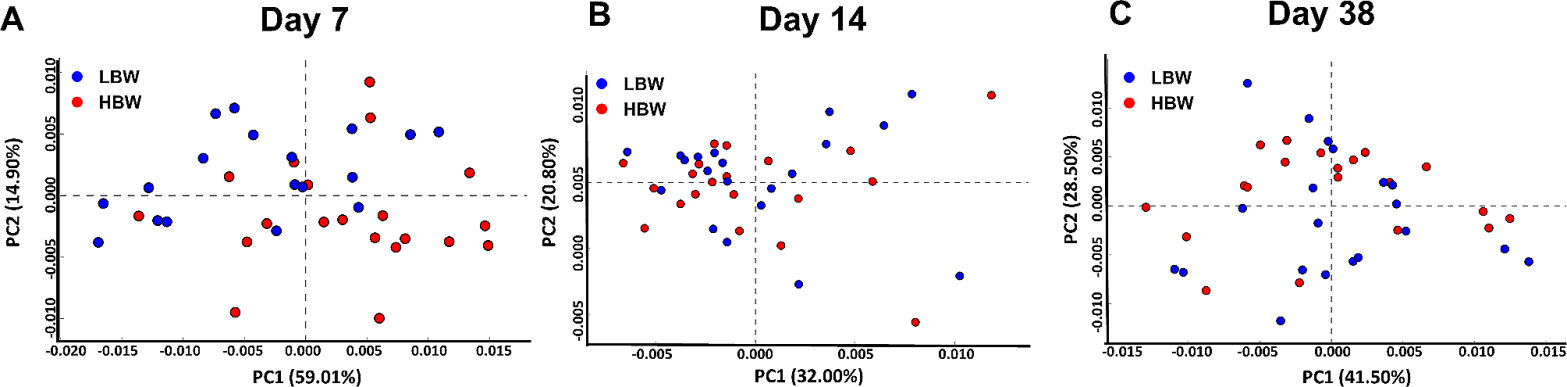
Principal component analysis of predicted pathways of the differential microbiota in low (LBW) and high (HBW) body weight groups on day 7 (**A**), day 14 (**B**), and day 38 (**C**)

**Fig. 9:**
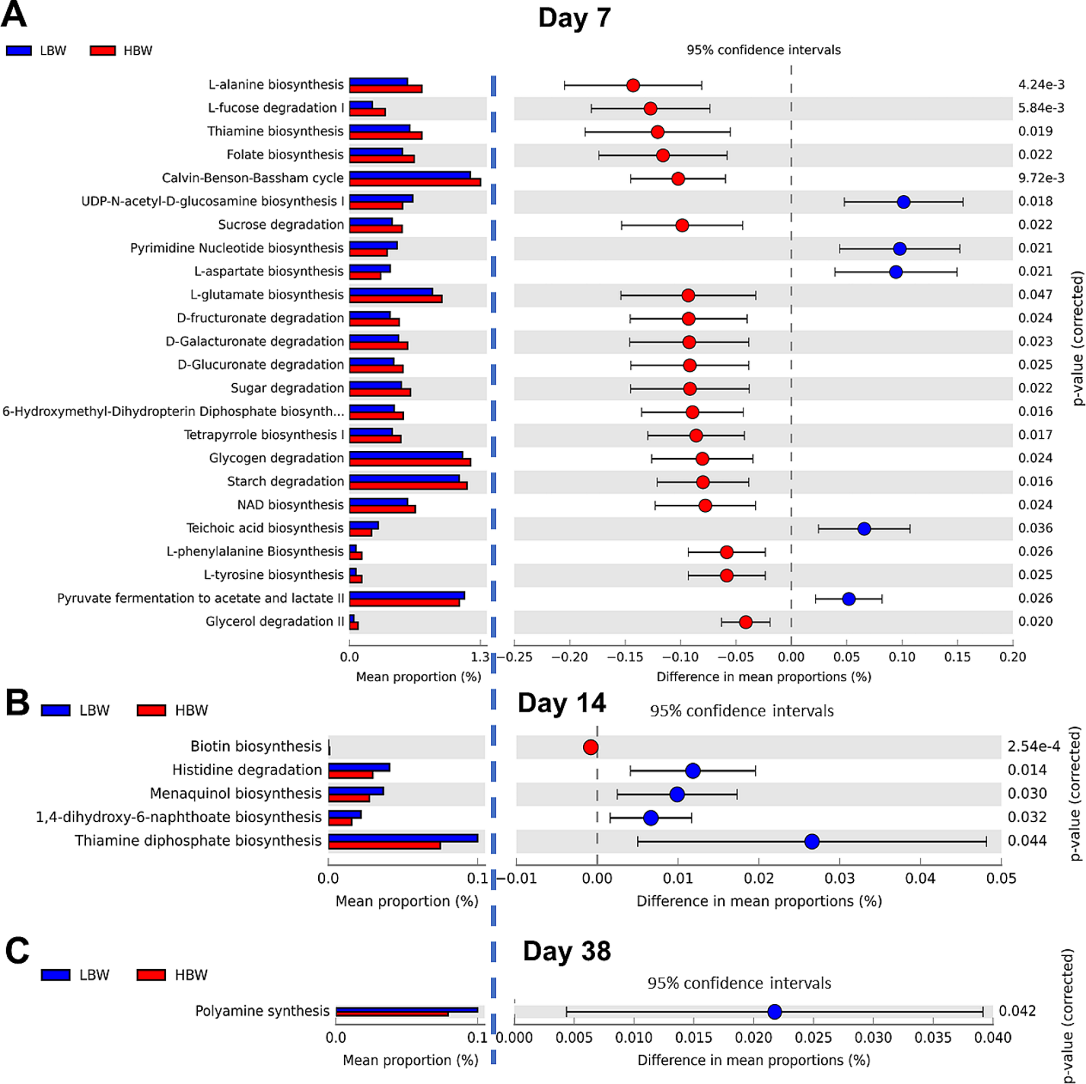
Predicted functions of the cecal microbiota of low (LBW) and high. (HBW) body weight broilers of both hatching systems (HS) on day 7 (**A**), day 14 (**B**), and day 38 (**C**). Only differentially regulated metabolic pathways are shown (FDR < 0.05)

The principal component analysis (PCA) of the metabolic pathways associated with caecal microbiota showed a clear separation between the LBW and HBW groups on day 7 (Fig. 8A), However, over time, the functional profiles of the microbiota converged across BW groups, as evidenced by the lack of distinct separation on days 14 and 38 (Fig. 8B and C). The PICRUST2 (Phylogenetic Investigation of Communities by Reconstruction of Unobserved States) output was analyzed by two-way ANOVA with FDR cut-off value of 0.05 and results revealed 25 significantly different microbial pathways on day 7 between the LBW and HBW groups (Fig. 9A). The LBW group showed enrichment in microbial pathways involved in the biosynthesis of cell wall components (UDP-N-acetyl-D-glucosamine, and teichoic acid), nucleotides (pyrimidine), and fermentation (lactate and butanoic acid). Microbial pathways enriched in the biosynthesis of amino acids (Thiamine, phenylalanine, tyrosine, and glutamate), cofactors (tetrapyrrole, NAD), and vitamins (folate) were higher in the HBW group compared to the LBW group. It is worth mentioning that the LBW group also demonstrated a higher relative abundance in one pathway of amino acid biosynthesis (aspartate). Moreover, bacteria of the HBW group exhibited enrichment in degradation pathways of simple and complex carbohydrates (fucose, starch, glycerol, sucrose, fructuronate, glucuronate, and other sugars) compared to the LBW group. On day 14, five microbial pathways were enriched in both LBW and HBW groups, primarily associated with cofactor synthesis (biotin, menaquinol, and 1,4-dihydroxy-6-naphthoate) and amino acid degradation (histidine, Fig. 9B). On day 38, only one function related to polyamine synthesis was enriched in LBW compared to HBW birds (Fig. 9C). PICRUST2 functional prediction analysis revealed 11 differentially abundant microbial pathways between HH and HOF groups on day 7, and 5 differential pathways on day 14, with no significant differences observed on day 38 (Additional file 1, Fig. S2 and S3). Interaction between HS and BW was observed solely on day 7, with the HOF-HBW group demonstrating a higher abundance of starch degradation and Calvin Benson Bassham cycle pathways compared to the other groups.

### Correlation of bacterial genera with body weight

**Fig. 10:**
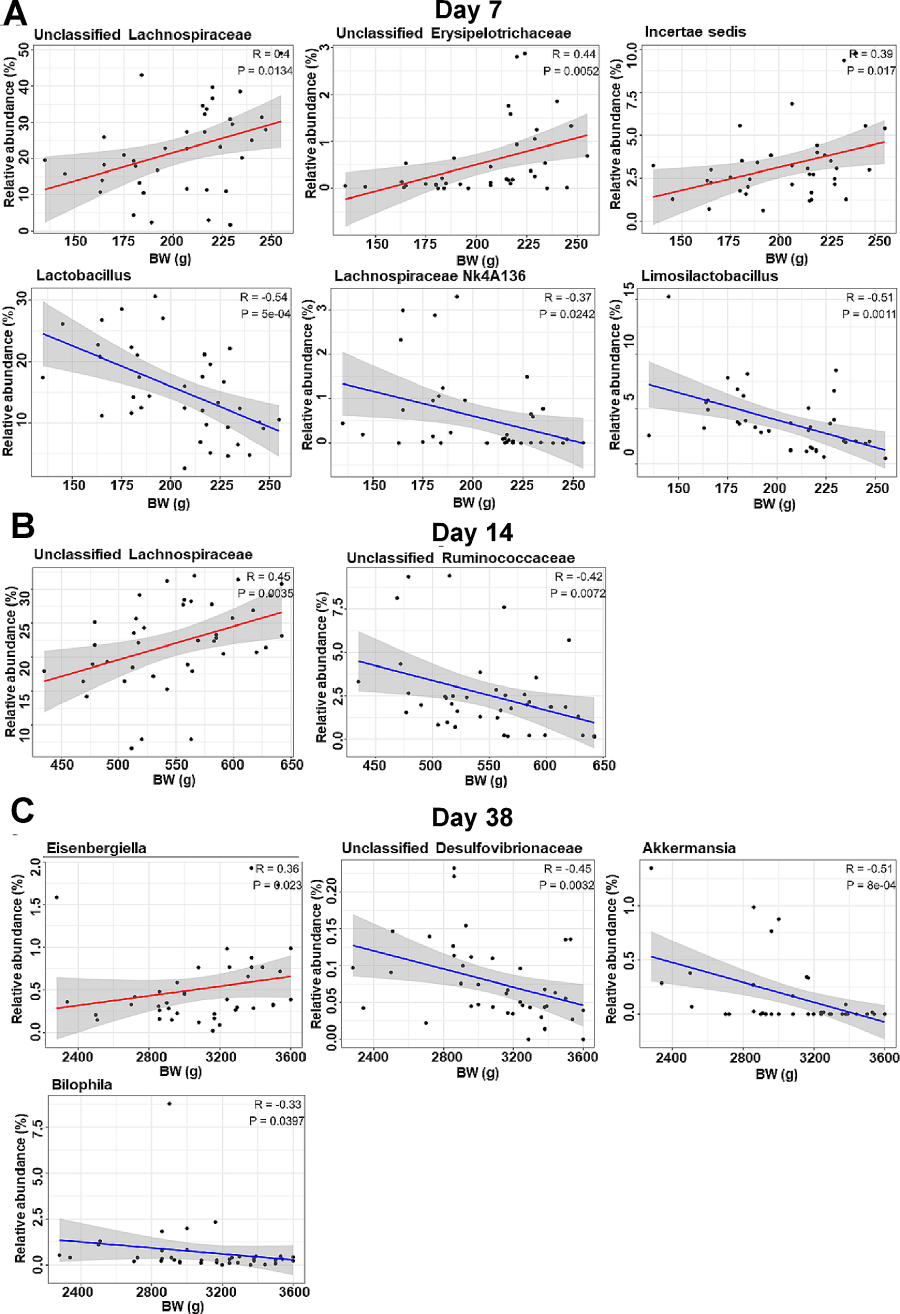
Spearman correlation between body weight (BW) and differentially abundant bacterial genera identified via LEfSe analysis in broiler chickens of both hatching systems (HS) on day 7 (**A**), day 14 (**B**), and day 38 (**C**). Only those features with a *P*-value less than 0.05 and an absolute correlation coefficient (|R|) greater than 0.30 are shown. The line of best fit is represented by a solid line (red = positive correlation, blue = negative correlation), while the gray shaded area around the line depicts the 95% confidence interval

To further identify the bacterial genera associated with BW, Spearman correlation analysis was performed using the genera differentially enriched based on the LEfSe results (Fig. 10). On day 7, 6 out of 12 genera showed significant correlations with BW, including unclassified *Lachnospiraceae*, unclassified *Erysipelotrichaceae* and *Incertae Sedis* positively correlated, and *Lactobacillus*, *Lachnospiraceae NK4136* and *Limosilactobacillus* negatively correlated (Fig. 10A). On day 14, 2 out of 9 genera exhibited correlations, with unclassified *Lachnospiraceae* favorably correlated and unclassified *Ruminococcaceae* negatively correlated with BW (Fig. 10B). On day 38, 3 out of 27 genera showed significant correlations with BW, including *Eisenbergiella* positively correlated, while *Akkermansia*, *Bilophila*, and unclassified *Desulfovibrionaceae* were negatively correlated with BW (Fig. 10C).

## Discussion

Our findings revealed variations in growth indices, VFA concentrations, and gut microbiome characteristics among broilers with different BW. HS resulted in transient effects on growth performance and exerted limited changes in caecal microbiota composition. Considering our initial hypothesis that HS might influence the investigated physiological mechanisms in broilers with varying BW and consequently affect their post-hatch growth patterns, we observed barely any interaction effect between HS and BW. Given the observed independent actions of HS and BW, we will present a separate discussion of these factors.

### Effect of hatching system

#### Growth performance

HOF had only short-term effects on broiler performance, with initial differences in weight between HH and HOF chicks disappearing within the first week. The HH chicks exhibited catch-up growth, likely due to their capacity to compensate for early-life feed and water deprivation experienced during the long hatch window and transportation [17, 21, 22]. Juul-Madsen et al. [23] reported compensatory growth in chicks deprived of feed for 24 h, reaching the weight of early-fed chicks by day 8, but chicks deprived for 48 h failed to reach similar weights even at 6 weeks of age. Similar to de Jong et al. [17], the relatively short duration of feed deprivation in the present study may not have been prolonged enough to induce significant and persistent effects on performance.

#### Microbiota composition

HS failed to demonstrate any effect on α and β diversity of gut microbiota in the present study, aligning with the findings of de Jong et al. [24], who observed no differences in diversity or composition of microbiota between HH and HOF chicks across all ages. However, our investigation did identify HS-dependent differences in caecal microbial communities across all time points (Fig. 5), with a noteworthy increase in the *Escherichia-Shigella* abundance in HH chicks on day 7 (Fig. 5A). The enrichment of these potentially pathogenic bacteria in HH chicks during early-life emphasize the importance of hatching environment, as they can potentially cause subclinical or clinical disease and impact the performance of broiler. Despite the HS-related microbial variations during the early stage, the microbiota community composition gradually converged over time, with few bacterial genera being different between HH and HOF chickens by slaughter age (Fig. 5C). Jong et al. [24] showed that broiler chicks subjected to different hatching conditions did not exhibit differences in their gut microbiota composition from the outset of the study. Similarly, Simon [25] reported ileal bacterial composition differences in broilers and laying hens fed immediately post-hatch versus those with feed deprivation for 72 h, but no significant differences persisted from day 21 onwards. This suggests that as the birds mature and undergo similar rearing conditions, the influence of the hatching environment and initial feeding time on the gut microbiome becomes less pronounced. Other factors, such as diet, housing conditions, BW, and age, likely take over and a exert stronger impact on shaping the gut microbial community composition.

### Effect of body weight

#### Growth performance

The LBW chicks were unable to overcome setbacks in weight throughout the study, even when reared under identical management conditions to their heavier counterparts (Table 1). The chickens in the HBW group exhibited higher ADG during the starter and grower phases. Although weight gains were similar between BW groups in the finisher phase, HBW birds maintained weight advantage due to their initial higher weight and faster early growth. These findings emphasize the significance of first-week weight on subsequent growth and slaughter weight of broilers as supported by literature showing a high positive correlation between chick weight at 7 days and harvest weight [26]. Consistent with a previous study on broilers [27], HBW chickens showed increased feed intake, possibly requiring more feed to sustain rapid growth. It further suggests that variations in feed intake since the initial days led to divergent weight gains, consequently impacting growth homogeneity directly.

#### Microbiota composition

Reduced microbial diversity was observed in the HBW group on days 7 and 38, consistent with certain studies [9, 28], even though some others suggest that HBW chickens might harbor more diverse bacteria than their LBW counterparts [14]. The LBW group demonstrated an age-dependent shift in microbiota development, initially harboring higher levels of immature and variable taxa, mainly comprising aerotolerant bacteria, such as most Enterobacteriaceae and Lactobacillaceae, which gradually transitioned to other microbial communities after day 14. This early microbiota profile suggests a less mature microbial composition in LBW chickens that evolves over time, leading to unstable microbiota communities and contributing to high species richness [29]. In contrast, the HBW group established microbial patterns typical of adult chickens from the outset, dominated by obligate anaerobic taxa from Firmicutes and Bacteroidetes including unclassified *Lachnospiraceae* and *Alistipes*. This age-dependent microbiota succession has been corroborated by previous studies, which found that newly-hatched chicks are initially dominated by rapidly colonizing bacterial groups like *Escherichia-Shigella* and *Streptococcus*, followed by a subsequent increase in the abundance of *Lactobacillus* from day 3 to day 14 of age [30]. Later in life, representatives from the phylum Bacteroidetes colonize and dominate the gut [31]. The early establishment of mature microbiota in HBW chickens may confer intestinal microbial stability and improved resilience to disturbances. Bilal et al. [32] reported that the presence of mature microbiota in day-old chicks can accelerate gut development, positively impacting overall health and productivity.

The unclassified *Lachnospiraceae* emerged as a biomarker in the HBW group, consistently enriched and strongly correlated with BW on days 7 and 14 (Fig. 6). *Lachnospiraceae* members are known for their ability to break down plant fibers and produce SCFAs, particularly butyrate, which promotes intestinal health, and host growth, and has immunomodulatory benefits [7, 33]. *Christensenellaceae R-7* was significantly higher in HBW chickens on day 7, previously found positively correlated with BW and muscle fiber diameter [34]. *Alistipes* was also recognized as biomarkers in the HBW group on days 7 and 38. Alistipes is an efficient colonizer of the caeca, promotes the growth of broiler chickens by producing SCFAs [35], and has been shown to be more abundant in HBW chicken [36]. The genera *Blautia* and *Subdoligranulum* were found as biomarkers in the HBW group on day 14. *Blautia* was previously identified in HBW broilers [14], and generates acetate by converting acetyl-CoA from pyruvate through the Wood-Ljungdahl pathway by fermenting both glucose and indigestible dietary fibers [37]. *Subdoligranulum* represents a sign of improved gut health as it produces SCFAs (i.e. butyrate) and influence gut physiology [38]. *Faecalibacterium*, a saccharolytic butyrate-producing bacterium, has been used as a probiotic in livestock [15], and emerged as a potential biomarker for enhanced performance in the later stages of life. *Eisenbergiella*, and the unclassified *Clostridia vadin BB60* group, capable of degrading complex plant polysaccharides like cellulose and hemicellulose [39], were prominent members of the gut microbiota in HBW chickens on day 38 compared to the LBW counterparts. *Flavonifractor* was also increased in HBW chickens [14], consistent with the previous study, and has been involved in SCFA production [40]. *Romboutsia* produces SCFAs, especially butyrate, which was enriched in the HBW group on day 14 and became abundant in the LBW group on day 38, previously positively associated with BW and ADG in broilers [41].

The enrichment in the phylum Proteobacteria and the genus *Escherichia-Shigella* on days 7 and 38 of the LBW group suggestes these genera to be potential biomarkers for lower BW in both early and later growth stages. The identified *Escherichia-Shigella* species in our study (*E. coli*,* S. boydii*,* S. dysenteriae*,* S. flexneri*, and *S. sonnei*) can be associated with colibacillosis and Shigellosis, leading to economic losses, reduced productivity, and compromised food safety [14]. Certain *Enterococcus* strains are intestinal commensals in farm animals, play crucial role in gut health, and are used as probiotics in poultry. However, some strains invade the intestinal mucosa and cause systemic infections [42]. Although *Enterococcus* species (*E. faecalis* and *E. faecium*) are often used as probiotics, their higher abundance in LBW chickens suggests that their presence does not always correlate with improved performance and may be associated with reduced productivity. Dolka et al. [43] reported that *E. faecalis* and *E. faecium* are sometimes considered opportunistic pathogens that can adversely affect growth in chickens under specific circumstances. *Streptococcus*, an opportunistic pathogen often causing secondary infections [44], was abundant in the LBW group. The LBW group also presented a higher abundance of the genus *Akkermansia*, previously linked to lower BW in broilers [9, 14]. Involved in mucin degradation, this genus is considered a biomarker for lipid metabolism and has been demonstrated to be beneficial in addressing obesity [45]. The genus *Bilophila* demonstrated a negative association with BW and has been reported in high abundance in intestinal diseases and inflammation in chickens [46]. This genus unclassified *Ruminococcaceae* on day 14 was found to be more enriched in the LBW group, which is in agreement with the observations of Farkas et al. [47]. Similarly, the pectin-degrading genus *Monoglobus* and the gut health-promoting genus *Lachnospiraceae NK4A136* group were also more abundant in LBW chickens. Although the precise mechanisms by which these bacteria influence LBW chickens are not fully understood, other factors such as feed intake or FCR may have a relevant impact on caecal microbiota besides BW, warranting further exploration. We found a negative correlation between *Lactobacillus* and BW, consistent with other studies linking this genus to decreased chicken productivity [12, 48]. *Lactobacilli* are highly dependent on the amino acids available in the small intestine [49]. The possibly reduced protein digestion and lower absorption capacity in the small intestine of LBW chicks may have increased protein bypass to the lower intestine, providing easily available substrates to *Lactobacilli*, and consequent activation of this microbial group in the caecum [50]. Some studies highlighted the implication of higher *Lactobacillus* levels with broiler growth reduction due to impaired fat absorption linked to the deconjugation of bile acids [51]. In our study, chicks categorized as LBW on day 7 were 21% lighter than the Aviagen target for male Ross 308 broilers and 22% lighter than HBW chicks in the study, reflecting the underperforming category typically can be observed in commercial settings. By day 38, both LBW and HBW groups exceeded expected Aviagen thresholds, with a 300 g (10%) difference between categories, which is a smaller gap than typically observed commercially at slaughter age [14]. Thus, our study primarily explained performance-related microbial biomarkers that more effectively account for the exceptional growth performance of HBW chicks, rather than emphasizing the factors contributing to poor performance in LBW birds.

#### Volatile fatty acid differences between BW groups

Most SCFAs showed significantly or numerically higher concentrations in the HBW group, while BCFAs were increased in the LBW group. SCFAs have been related to BW changes, with elevated acetate levels observed in overweight human individuals [52]. The BCFAs are generated through protein fermentation in the cecum and are often associated with unfavorable shifts in the microbial community and increased ammonia production [53]. Specific bacterial genera enriched in LBW chickens, including *Negativibacillus* and *Escherichia-Shigella*, positively correlated with BCFA isobutyrate on days 14 and 38, respectively, aligning with prior study [54] linking *Escherichia-Shigella* abundance to cecal isobutyrate concentration. Conversely, in the HBW group, *Blautia* abundance positively correlated with acetate on day 14, while *Flavonifractor* enrichment on day 38 showed positive correlations with acetate, butyrate, caproate, and total SCFA concentrations. These observations suggest that specific bacteria in each BW category can produce certain types of VFAs, which could influence intestinal health in a BW-dependent manner.

### Predicted function of caecal microbiota

During earlier days, lower taxonomic but higher functional differences existed between LBW and HBW microbiota. By day 38, taxonomic differences of microbiota increased while functional differences decreased suggesting microbiota in both groups were likely fulfilling similar functional roles in later stages. Early colonizers demonstrated greater versatility and metabolic activity compared to the late colonizers, corroborating a previous study on infants where microbiota at 1 month of age were more functionally active and independent compared to 6 months [55]. The early life differences between BW groups resulted in higher positive microbial functionalities in HBW chickens, allowing them to have an initial performance boost, resulting in faster growth, finally reaching an equilibrium on day 38 (Fig. 9). The HBW group exhibited enrichment of microbial genes involved in biosynthesis pathways (amino acids, cofactors, and vitamins). It is speculated that this might have contributed to their better performance as previous studies have reported an association between microbial functions related to nutrient biosynthesis and increased weight gain [10]. The higher feed intake observed in HBW chickens suggests that a greater quantity of feed components, which would otherwise be indigestible by the host, likely reached the ceca for microbial utilization and fermentation. The HBW group possessed a gut microbiota better adapted to utilizing both complex and simple carbohydrates, potentially producing essential nutrients including SCFAs, thereby facilitating rapid weight gain. The LBW group exhibited enrichment in the microbial pathway for pyruvate fermentation to acetate and lactate. The higher abundance of *Lactobacillus*, which ferments pyruvate into lactate, may negatively impact mucosal barrier function and host health [56], yet metabolic cross-feeding enables lactate-utilizing bacteria to convert it into other metabolites [57]. LBW group also exhibited enrichment in the microbial pathway responsible for UDP-N-acetyl-D-glucosamine biosynthesis, a precursor for cell wall peptidoglycan, lipopolysaccharide, and the enterobacterial common antigen, as observed in *Escherichia coli* [58].

Predicting functional activities based solely on taxonomic composition or genomic data may not fully reflect the dynamic and context-dependent nature of microbial metabolism. To address these limitations, future studies are suggested to complement 16 S rRNA gene amplicon sequencing with metatranscriptomics or metabolomics approaches, which can provide more direct and comprehensive insights into the functional potential and metabolic activities of the gut microbiome.

### Interaction effect of HS and BW on Microbiota

Previous studies have suggested that variations between low and high-weight birds might originate before their placement in the barn [14], influenced by factors like hatching environment, chick transportation, and access to first feed. The interaction between HS and BW showed no impact on α and β diversities (Fig. 1). Nevertheless, some initial interactions between HS and BW were noted concerning early-life microbiota composition, but these interactions markedly decreased over time (Additional file 1, Fig. S1). Our findings indicate that factors associated with the hatching conditions do not have long-term impact on BW-related microbiota characteristics of birds. Instead, selection by the host (i.e. BW of birds) emerged as a more potent driver for shaping the intestinal microbiota, overshadowing the effects of hatching conditions.

### Conclusions

We observed that HS had only short-lasting effects on chicken performance and microbiota composition, and barely showed an impact on BW-related differences in the variables investigated. The disparities in growth among broilers were primarily driven by the bird’s initial BW, rather than the hatching conditions. A higher BW in the first week allows chicks to maintain an advantage over the chicks with a lower BW, shaping differences in feed intake and microbiota characteristics, and subsequently influencing overall performance. SCFAs (which are beneficial) were higher in the HBW group and BCFAs (which are unfavorable) were higher in the LBW group. Genera like unclassified *Lachnospiraceae* early on, and *Faecalibacterium* and *Clostridia vadin BB60* group in later growth stages could serve as biomarkers for enhanced performance in broilers. Conversely, *Escherichia-Shigella* and *Streptococcus* appear to be a biomarker for suboptimal performance during early and later growth stages. The HBW group demonstrated enrichment of gut-health-promoting taxa, which may have contributed to enhanced performance through various mechanisms such as better utilization of feed, enhanced metabolic activity, biosynthesis of essential nutrients, production of energy-rich metabolites, and modulation of the immune system. Our study further strengthens the understanding regarding the microbiota characteristics that impact broiler performance across growth stages under uniform rearing conditions. These findings provide potential insights for developing strategies to modulate and establish a more uniform and beneficial microbiota in underperforming broilers, thereby ensuring greater uniformity.

## Methods

### Animals, housing and management

This study involved Ross 308 male chicks, sourced from eggs intended for both HS and originating from the same 40-week-old parent flock. These eggs were obtained from Hatchery Belgabroed N.V. (Merksplas, Belgium). The HOF system involved obtaining fertilized eggs and transporting them to the barn after candling on embryonic day 18. The eggs were placed on the wood shavings at optimal housing conditions with regulated eggshell temperatures (36.1–37.2 °C) to support optimal embryonic development. Chicks started hatching on embryonic day 19. Once 75% of the chicks had hatched, the primary focus shifted from regulating the eggshell temperature to maintaining the chicks’ body temperature between 39.5 and 40.5 °C. Chicks were provided 24 h of light to facilitate their immediate access to feed and water upon hatching. The HH chicks hatched in a hatchery (Belgabroed N.V., Belgium) under standard procedures. The hatch window typically lasts 24–36 h, after which chicks were removed from the hatcher [59]. Following grading, sexing, and other processes, chicks were transported 108 km to the farm, which took approximately 2 h. Consequently, for some chicks, it was more than 40 h before placing into the pens and accessing feed and water, considering the hatch window, hatchery protocols, and transportation time.

Following standard commercial practices, the day of arrival of HH chicks at the broiler house was designated “day 1” for both HS. On this day, HOF chicks underwent manual grading and sexing, including culling of chicks with deformities. By the end of day 1, the temperature of the barn was set at 33 °C, gradually decreasing by approximately 0.5 °C daily until it reached 21.5 °C on day 21, remaining constant for the remainder of the experiment. Birds were reared on a concrete floor with wood shavings as bedding material, provided with one hour of darkness on day 1, increasing to six hours from day 7 onwards. They had unrestricted access to water and received three-phase commercial diets (starter, grower, and finisher) without exposure to antibiotics (Table 3).

**Table 3:** Composition (%) of the feed offered to broilers during starter, grower, and finisher phases

Ingredients %	Starter 1–14 days	Grower 15–28 days	Finisher 29–38 days
Maize	15.0	10.0	5.0
Wheat (fine)	33.73	39.20	48.88
Wheat (coarse)	5.0	10.0	10.0
Maize gluten	2.88	3.32	0
Soy oil	4.61	5.02	5.32
Soybean meal	29.86	24.79	25.42
Sunflower meal	2.5	2.5	0
Oat hull (coarse)	1.0	0	0
Sodium bicarbonate	0.23	0.23	0.25
Salt	0.16	0.16	0.16
Choline 75%	0.09	0.09	0.09
^1^Premix	0.30	0.30	0.30
Limestone	1.44	1.31	1.19
Monocalcium Phosphate	1.13	0.92	0.70
Lysine	0.55	0.52	0.30
Methionine	0.26	0.22	0.22
L-Threonine	0.14	0.12	0.09
L-Valine	0.02	0	0
^2^Avi-Deccox	0.05	0.05	0
L-Arginine	0.03	0.02	0.02
Palm oil spray	1.0	1.22	2.03
^3^Phytase	0.01	0.01	0.01
Chemical Composition			
Metabolizable energy (kcal/kg)	3000	3100	3200
Digestible lysine (%)	1.28	1.15	1.02
Crude protein (%)	23.0	21.5	19.5
Calcium (%)	0.96	0.87	0.78
Available phosphorus (%)	0.48	0.44	0.39
Sodium (%)	0.14	0.14	0.14
Chloride (%)	0.18	0.18	0.16
Potassium (%)	1.0	0.90	0.90

^1^Provided per kg feed: Vit A 10.0 IU, Vit D3 2750 IU, 25-hydroxycholecalciferol 0.06 mg, Vit E 90 mg, Copper 15 mg, Iron 15 mg, Manganese 85 mg, Zinc 50 mg, Iodine 2 mg, Selenium 0.4 mg

^2^Provided per kg feed: 30.3 mg decoquinate

^3^Provided per kg feed: 500 FTU

### Study design

**Fig. 11:**
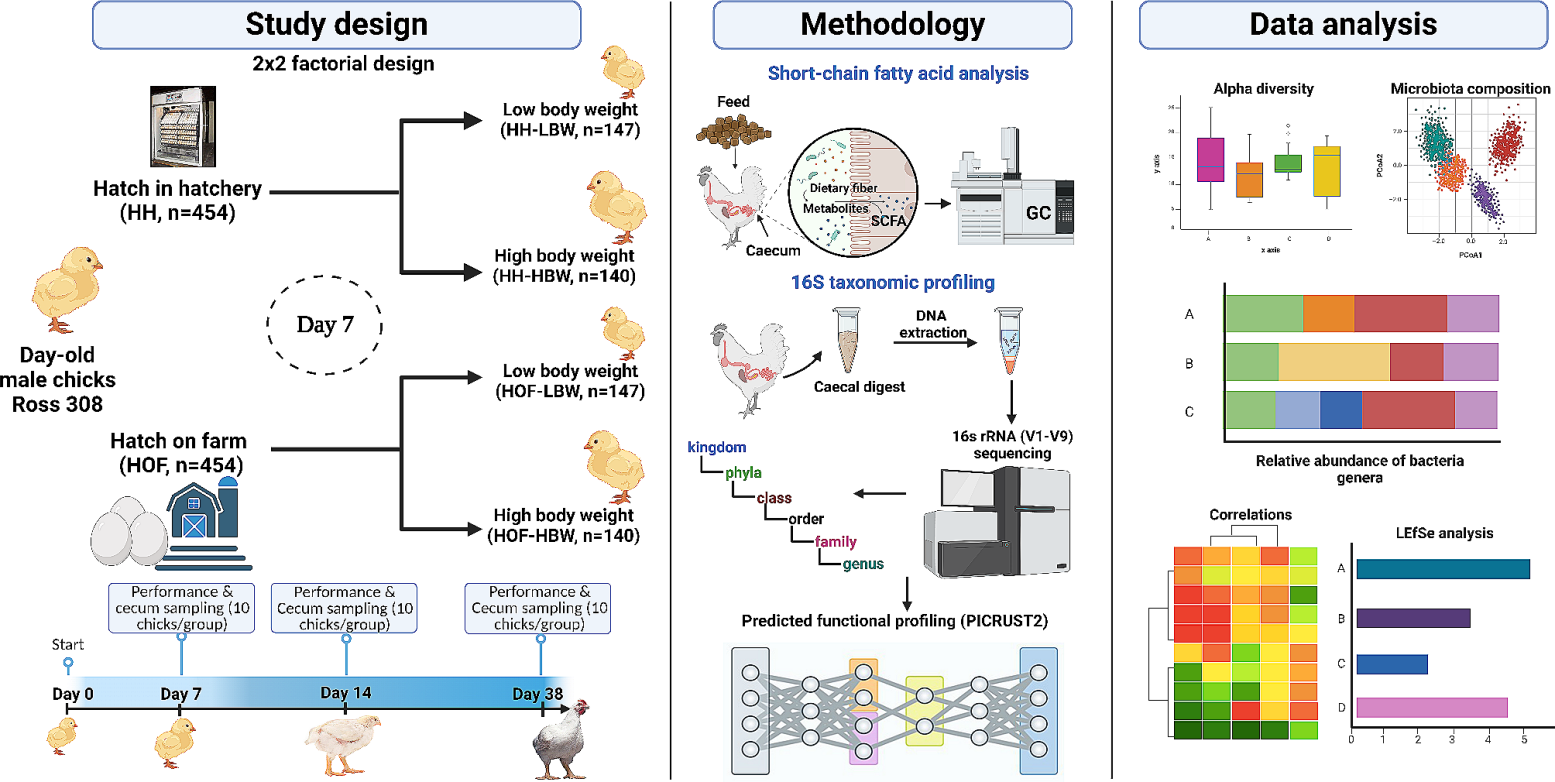
Flow chart of the study design, timeline, and parameters investigated. This image was created with Biorender.com

The study included 908 day-old male Ross 308 broiler chicks, 454 of which were from each of the two HS (Fig. 11). For each HS, chicks were co-reared until day 7, and then grouped into BW categories as follows: low (LBW, *n* = 147), birds falling below the mean BW by half the standard deviation (½ × SD); middle (*n* = 167), birds within the mean BW and ± ½ × SD; and high (HBW, *n* = 140), birds surpassing the mean BW by half the SD (½ × SD). The middle BW birds were excluded from the study. The study design was a 2 × 2 factorial arrangement, investigating two main factors: HS (HH vs. HOF) and BW (LBW vs. HBW), and their interaction (HS × BW). The chicks were reared in the same management conditions following the commercial stocking density limits, housed in 28 pens ((1.3m^2^/pen, 7 replicate pens per experimental group) of LBW (*n* = 21/pen) and HBW (*n* = 20/pen). The LBW pens each had one extra bird so as to reach a similar stocking density to that of the HBW pens.

### Growth performance

Birds were weighed individually on days 0, 7, 14, 28, and 38 post-hatch and feed intake was recorded per pen on days 7, 14, 28, and 38. Mortalities and postmortem weight were recorded for the calculation of ADG, ADFI, and mortality-corrected FCR. The CV (%) for weight uniformity in each group was calculated on days 7, 14, 28, and 38 by taking the ratio of the standard deviation (SD) to the mean BW and multiplying by 100.

### Chick sampling

On days 7, 14, and 38, ten birds from each experimental group were killed by electronarcosis followed by decapitation for sampling purposes. Digesta samples were carefully collected from both caeca, placed in 2 mL vials, snap-frozen, and stored at -80 °C until further analysis of microbiota and VFAs.

### Volatile fatty acid analysis

The level of short- (SCFAs; acetate, propionate, butyrate, valerate, and caproate) and branched- (BCFAs; isobutyrate, isovalerate, and isocaproate) fatty acids were determined using a method previously detailed by Van Craeyveld et al. [60] with minor modifications. Briefly, 450–500 mg caecal content was mixed with 100 µl of a 2-methylhexanoic acid, followed by the addition of 200 µL of 25% NaCl solution and 9.2 M sulfuric acid each. Subsequently, 800 µL diethyl ether was added to extract organic acids, followed by centrifugation at 3800 × g for 5 min at 4 °C. The resulting supernatants were transferred to a reactive vial containing 0.2–0.3 g of activated anhydrous sodium sulfate and centrifuged at 3800 × g for 6 min at 4 °C before analysis. VFAs were quantified by gas chromatography on an HP 6890 Series GC System. This system had an Automatic Liquid Sampler (7683 Series Injector, Agilent Technologies) for cool on-column injection, a flame ionization detector, and a DB-FFAP capillary column (Agilent J&W GC Columns, 30 m length, 0.32 mm internal diameter, 0.25 μm film thickness). Nitrogen served as the carrier gas flowing at a 25 mL/min rate. The column temperature was maintained at 130 °C, while the injector and detector temperatures were set to 195 °C.

### DNA extraction and 16 S rRNA gene amplicon sequencing

DNA was extracted from approximately 250 mg of caecal digesta for 16 S rRNA gene markers using the QIAamp PowerFecal Pro DNA Kit (Qiagen Benelux B.V., Venlo, the Netherlands) in accordance with the manufacturer’s standard protocol. The concentration of obtained DNA was determined using a Nanodrop 2000 spectrophotometer (Thermo Fisher Scientific, Waltham, MA), while quality was assessed by 1% agarose gel electrophoresis. The full-length (V1-V9) 16 S rRNA gene was amplified via PCR using the universal primers 27 F: AGRGTTYGATYMTGGCTCAG and 1492R: RGYTACCTTGTTACGACTT, with sample-specific PacBio barcode sequences added. A ZymoBIOMICS™ Microbial Community DNA Standard (P/N: D6306, Lot ZRC190811) containing genomic DNA from six phylogenetically diverse bacteria was used as a positive control, and DNA from ultrapure water was used as a negative control. DNA libraries were generated from the amplified DNA, and sequencing was performed using the PacBio platform by the VIB Nucleomics Core (Leuven, Belgium).

### Sequence processing workflow

After sequencing, further data analysis was performed in R (v4.2.3, R Foundation, Vienna, Austria). The raw sequence data obtained from PacBio long-read amplicon sequencing underwent additional processing steps, including quality filtering, denoising, and removal of chimeric sequences, following the established long-read workflow by Callahan et al. [61]. After filtering and denoising, ASVs were inferred using the DADA2 R package. The ASVs were taxonomically classified by comparing them against the SILVA database (release 138) at a 99% shared identity using the Naive Bayes Classifier method. Downstream analysis focused on bacterial domain sequences, and positive control was excluded from the analysis, as it was included to verify the accuracy of the taxonomic assignment. Reads were decontaminated based on the negative control, which identified *Bradyrhizobium elkanii*, unclassified *0319-6G20*, and unclassified *Acidibacter* spp. as contaminants, and these were removed from the ASV table accordingly, resulted in 2776 ASVs on day 7, 2839 ASVs on day 14, and 4118 ASVs on day 38. The α-diversity and β-diversity were calculated in R using the phyloseq package (v1.40.0). For α-diversity, the rarefaction of the ASV table was performed to the minimum sample depth. Three α-diversity indices were calculated: Chao1, Shannon, and Simpson, which indicate microbial richness, overall diversity, and evenness, respectively. Two-way analysis of variance (ANOVA) was used for each α-diversity measure to compare the effects of HS, BW, and their interaction. β-diversity was determined using the Bray-Curtis dissimilarity matrix, which was obtained from the distance function in phyloseq and visualized via principal coordinate analysis (PCoA). Multivariate effects of HS and BW on β-diversity were evaluated by non-parametric permutational multivariate analysis of variance (PERMANOVA) using the adonis2 function with 999 permutations from the vegan package (v2.6.4). The differential abundance of caecal microbiota was calculated using LEfSe in R using the microbiome package (v1.18.0). The default parameter of LDA ≤ 2 was used with a significance threshold of *P* < 0.05. The obtained *P*-values were further adjusted FDR through the Benjamini-Hochberg method, with a stringent criterion of FDR < 0.05. The results were then visually represented based on the Log10 (LDA score). PICRUSt2 was used to predict the functional capabilities of the microbial communities in the different BW groups. This functional profiling was derived from the 16 S rRNA gene sequences and utilized the MetaCyc Metabolic Pathway Database as a reference [62]. The data obtained from PICRUST2 was analyzed through two-way ANOVA with FDR < 0.05. PCA, an unsupervised pattern recognition method, was used in R using the factoextra (v 1.0.7) package, to provide an overview of the predicted function data patterns between HS-BW groups.

## Statistical analysis

Shapiro-Wilk’s test in R was performed to evaluate the normality of data. Following the confirmation of normality, the BW data on day 1 for HH and HOF chicks was analyzed by Student’s *t-*test. The data on growth performance and VFA from day 7 onward were used to conduct the two-way ANOVA and Tukey’s post hoc test. The HS and BW were used as the fixed effects and the pens were considered as a random effect to account for potential confounding variation due to pen location and differing numbers of birds per pen. For all statistical tests, a *P*-value threshold of 0.05 was used to determine statistical significance, while a *P*-value between 0.05 and 0.10 indicated a trend toward significance. Spearman correlation analysis was performed in R using the psych package (v2.3.12) to determine the correlation between LEfSe-identified abundant bacterial genera and the BW and caecal VFA of broilers.

### Electronic supplementary material

Below is the link to the electronic supplementary material.

Supplementary Material 1

## Data Availability

The 16 S rRNA gene sequencing data can be accessed under Bioproject accession PRJNA1103836 at the NCBI website.

## Abbreviations

ADFI: Average Daily Feed Intake
ADG: Average Daily Gain
ANOVA: Analysis of Variance
ASV: Amplicon Sequence Variant
BCFA: Branched-Chain Fatty Acid
BW: Body Weight
CV: Coefficient of Variation
FCR: Feed Conversion Ratio
FDR: False Discovery Rate
HBW: High BW
HH: Hatchery- Hatched
HOF: Hatching On -Farm
HS: Hatching System
LBW: Low BW
LEfSe: Linear Discriminant Analysis Effect Size
PCoA: Principal Coordinate Analysis
PERMANOVA: Permutational Multivariate Analysis Of Variance
Picrust2: Phylogenetic investigation of communities by reconstruction of unobserved states
SCFA: Short-Chain Fatty Acid
SD: Standard Deviation
VFA: Volatile Fatty Acid
