# Supplementary material for: Assessing the impact of hatching system and body weight on the growth performance, caecal short-chain fatty acids, and microbiota composition and functionality in broilers
Source: Anim Microbiome. 2024 Jul 24;6:41. doi: 10.1186/s42523-024-00331-6 (PMC11271025; doi:10.1186/s42523-024-00331-6)
Supplement: Supplementary file 1 — Supplementary Material 1 [file 42523_2024_331_MOESM1_ESM.docx]

**Assessing the impact of hatching system and body weight on the growth performance, caecal short-chain fatty acids, and microbiota composition and functionality in broilers**

Muhammad Zeeshan Akram^1,2^, Ester Arévalo Sureda^1^, Luke Comer^1^, Matthias Corion^1^, Nadia Everaert^1, ͳ^

^1^Nutrition and Animal-Microbiota Ecosystems Laboratory, Department of Biosystems, KU Leuven, 3000-Heverlee, Belgium

^2^Precision Livestock and Nutrition Unit, Gembloux Agro-Bio Tech, University of Liège, Gembloux, Belgium,

^ͳ^Corresponding author: Nadia Everaert: [nadia.everaert@kuleuven.be](mailto:nadia.everaert@kuleuven.be)

**Table S1:** Relative abundance (%) of phyla in caecal samples of low (LBW) and high (HBW) body weight (BW) chickens hatched in the hatchery (HH) or on farm (HOF).

| Phylum | ^1^HS | | BW |  | ^2^Interaction | | | | SD |
| --- | --- | --- | --- | --- | --- | --- | --- | --- | --- |
|  | HH | HOF | LBW | HBW | HH-LBW | HH-HBW | HOF-LBW | HOF-HBW |  |
| Day 7 |  |  |  |  |  |  |  |  |  |
| Firmicutes | 99.7 | 99.7 | 99.6 | 99.8 | 99.6 | 99.8 | 99.7 | 99.7 | 0.47 |
| Proteobacteria | 0.23 | 0.19 | 0.32 | 0.11 | 0.39 | 0.08 | 0.25 | 0.13 | 0.289 |
| Bacteroidota | 0.03 | 0.04 | 0.00 | 0.07 | 0.00 | 0.05 | 0.00 | 0.08 | 0.333 |
| Others | 0.04 | 0.05 | 0.05 | 0.04 | 0.04 | 0.04 | 0.05 | 0.04 | 0.022 |
| Day 14 |  |  |  |  |  |  |  |  |  |
| Firmicutes | 97.4 | 97.6 | 97.3 | 97.7 | 97.2 | 97.5 | 97.3 | 97.8 | 1.27 |
| Bacteroidota | 2.18 | 1.93 | 2.24 | 1.87 | 2.38 | 1.98 | 2.11 | 1.76 | 1.060 |
| Proteobacteria | 0.26 | 0.23 | 0.26 | 0.22 | 0.27 | 0.25 | 0.25 | 0.20 | 0.435 |
| Others | 0.21 | 0.26 | 0.24 | 0.23 | 0.16 | 0.26 | 0.32 | 0.2 | 0.130 |
| Day 38 |  |  |  |  |  |  |  |  |  |
| Firmicutes | 93.8 | 94.8 | 93.7 | 94.9 | 93.7 | 93.9 | 93.8 | 95.9 | 3.66 |
| Cyanobacteria | 2.70 | 2.30 | 2.99 | 2.02 | 2.71 | 2.69 | 3.27 | 1.34 | 3.010 |
| Bacteroidota | 1.62 | 1.36 | 1.33 | 1.65 | 1.40 | 1.83 | 1.25 | 1.47 | 0.902 |
| Proteobacteria | 1.37 | 0.94 | 1.43 | 0.89 | 1.69 | 1.05 | 1.16 | 0.73 | 1.206 |
| Others | 0.48 | 0.6 | 0.58 | 0.5 | 0.48 | 0.47 | 0.65 | 0.53 | 0.201 |

^1^HS: hatching system

^2^HH-LBW: hatchery hatched low BW group (n = 10), HH-HBW: hatchery hatched high BW group (n = 10), HOF-LBW: hatched on-farm low BW group ((n = 10), HOF-HBW: hatched on-farm high BW group ((n = 10).

Data presented as mean and pooled standard deviation (SD).


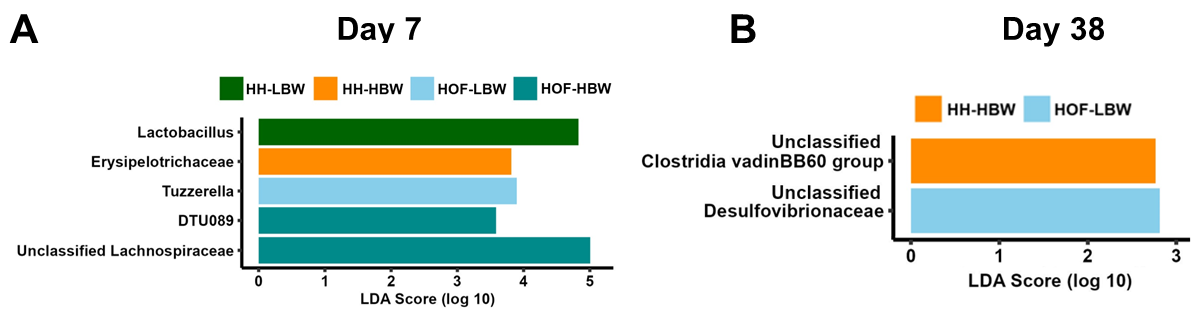


**Fig. S1**: Bacterial genera with significant interaction between hatching systems (HS) and body weight (BW) for differential analysis with LEfSe on day 7 (**A**) and day 38 (**B**). Only genera with FDR ≤ 0.05 and with an absolute value of LDA > 2 are presented.


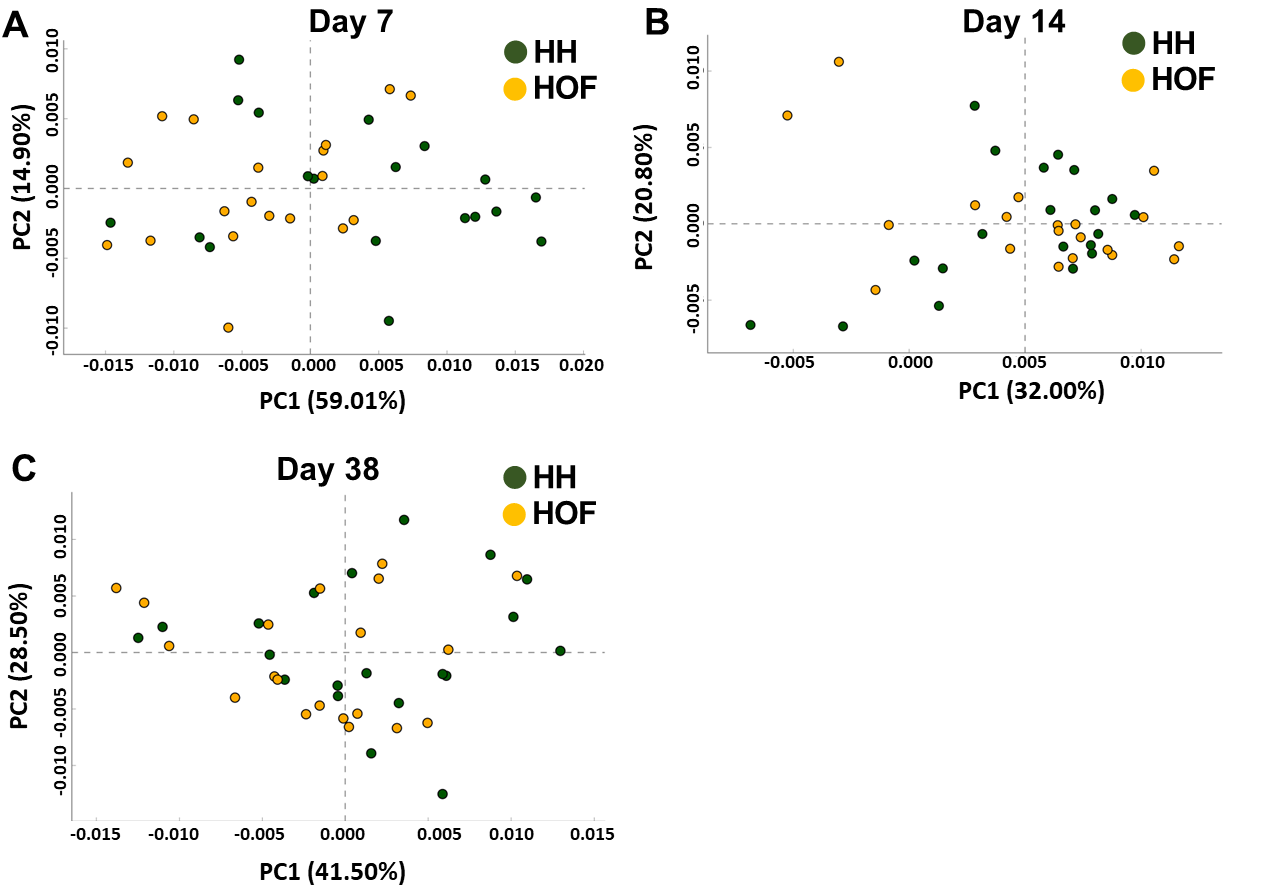
**Fig. S2:** Principal component analysis of predicted pathways of the differential microbiota in chicks hatched in the hatchery (HH) or on-farm (HOF) on day 7 (**A**), 14 (**B**), and 38 (**C**).


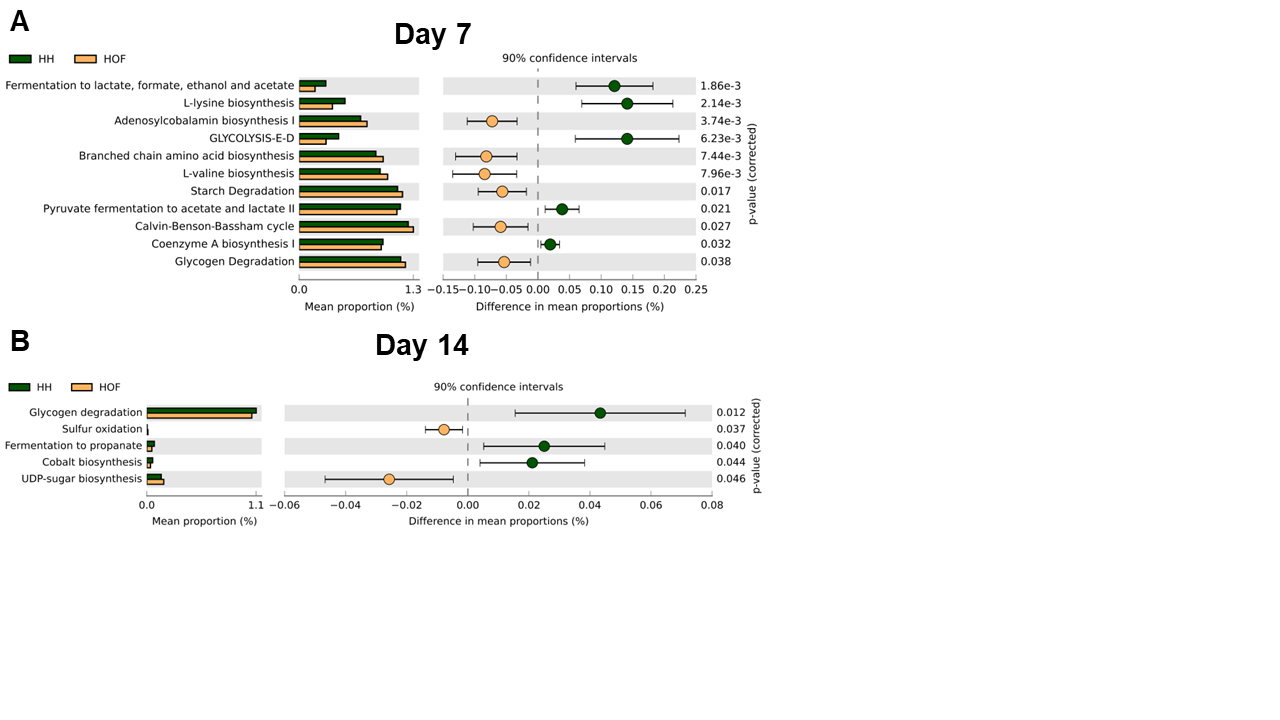


**Fig. S3:** Predicted functions of the cecal microbiota of broilers hatched in hatchery (HH) or on-farm (HOF) on day 7 (**A**) and day 14 (**B**). No differences were observed between HH and HOF chicks on day 38.
